# Targeted therapies prime oncogene-driven lung cancers for macrophage-mediated destruction

**DOI:** 10.1172/JCI169315

**Published:** 2024-03-14

**Authors:** Kyle Vaccaro, Juliet Allen, Troy W. Whitfield, Asaf Maoz, Sarah Reeves, José Velarde, Dian Yang, Anna Meglan, Juliano Ribeiro, Jasmine Blandin, Nicole Phan, George W. Bell, Aaron N. Hata, Kipp Weiskopf

**Affiliations:** 1 Whitehead Institute for Biomedical Research, Cambridge, Massachusetts, USA.; 2 Dana-Farber Cancer Institute, Boston, Massachusetts, USA.; 3 Massachusetts General Hospital Cancer Center, Boston, Massachusetts, USA.; 4 Department of Medicine, Massachusetts General Hospital and Harvard Medical School, Boston, Massachusetts, USA.; 5 Koch Institute for Integrative Cancer Research, Massachusetts Institute of Technology, Cambridge, Massachusetts, USA.

**Keywords:** Oncology, Therapeutics, Cancer immunotherapy, Lung cancer, Macrophages

## Abstract

Macrophage immune checkpoint inhibitors, such as anti-CD47 antibodies, show promise in clinical trials for solid and hematologic malignancies. However, the best strategies to use these therapies remain unknown, and ongoing studies suggest they may be most effective when used in combination with other anticancer agents. Here, we developed an unbiased, high-throughput screening platform to identify drugs that render lung cancer cells more vulnerable to macrophage attack, and we found that therapeutic synergy exists between genotype-directed therapies and anti-CD47 antibodies. In validation studies, we found that the combination of genotype-directed therapies and CD47 blockade elicited robust phagocytosis and eliminated persister cells in vitro and maximized antitumor responses in vivo. Importantly, these findings broadly applied to lung cancers with various RTK/MAPK pathway alterations — including *EGFR* mutations, *ALK* fusions, or *KRAS^G12C^* mutations. We observed downregulation of β2-microglobulin and CD73 as molecular mechanisms contributing to enhanced sensitivity to macrophage attack. Our findings demonstrate that dual inhibition of the RTK/MAPK pathway and the CD47/SIRPa axis is a promising immunotherapeutic strategy. Our study provides strong rationale for testing this therapeutic combination in patients with lung cancers bearing driver mutations.

## Abstract 2

Unbiased drug screens identify targeted therapies as drugs that make lung cancers with driver mutations more vulnerable to macrophage attack.

## Graphical abstract

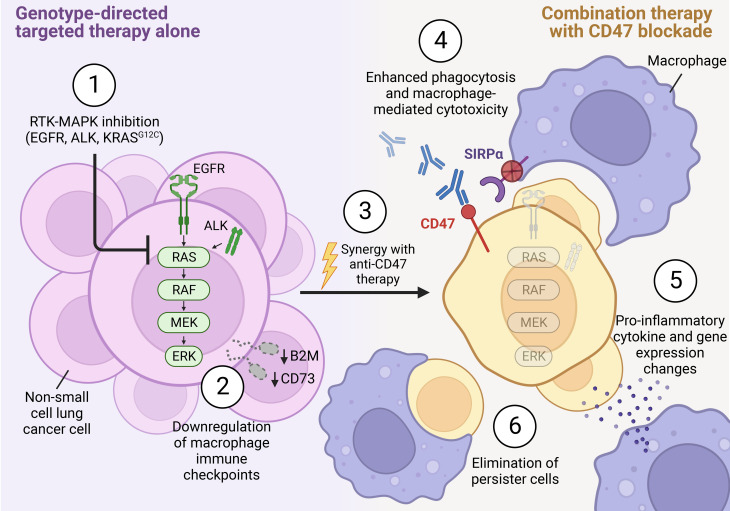

## Introduction

Many cancers arise from a single mutation in a proto-oncogene that drives tumor growth. These cancers are often treated with genotype-directed “targeted therapies,” small-molecule inhibitors that specifically disable growth signals from oncogenic driver mutations. These drugs are most frequently used to treat non–small cell lung cancer (NSCLC), in which up to 50% of patients may have an actionable mutation that can be targeted with specific small-molecule inhibitors (1). *EGFR* and *KRAS* mutations are among the most common actionable mutations in lung cancer (1–4). Although targeted therapies extend patients’ lives, they typically do not cure patients and responses generally last for only a few years (2, 3, 5). For these reasons, there is a critical need to identify more effective ways to treat lung cancers with oncogenic driver mutations.

Based on the success of cancer immunotherapy, an appealing strategy has been to combine targeted therapies with agents that activate the immune system to attack cancer. However, lung cancers with driver mutations may have lower tumor mutational burden and fewer neoantigens, thereby impairing adaptive immune responses (6, 7). Furthermore, the combination of some targeted therapies with anti–PD-1/PD-L1 agents has been limited in efficacy or produced synergistic toxicity (8). In contrast to targeting T cells, macrophages are an attractive alternative target, because they are often the most common infiltrating immune cell within the tumor microenvironment (9, 10). Nevertheless, the combination of targeted therapies with macrophage-directed therapies is relatively unexplored.

The CD47/SIRPa interaction is the best-characterized immune checkpoint that controls macrophages in tumors (11). CD47 is highly expressed on many cancers, and it binds to the inhibitory receptor SIRPa that is present on macrophages and other myeloid cells. When CD47 binds to SIRPa, it sends inhibitory signals to the macrophage that prevent phagocytosis. Therapies that block the CD47/SIRPa axis stimulate macrophage engulfment and destruction of cancer cells in preclinical models and show encouraging signs of efficacy in ongoing clinical trials for solid and hematologic malignancies (11–15). However, in many cases, blockade of CD47 by itself is not sufficient to induce macrophage antitumor responses, but instead it lowers the threshold for macrophage activation in the presence of a second stimulus (16). Thus, CD47-blocking therapies may work best in combination with other therapies that make cancer cells more vulnerable to macrophage attack (16–18). At present, the best combination modalities remain unknown, and unbiased screening efforts have not been performed to rationally identify drugs that synergize with anti-CD47 agents or other macrophage-directed therapies.

In this study, we aimed to discover novel therapeutic strategies to sensitize lung cancer cells to macrophage-mediated destruction. We developed a high-throughput screening platform to measure antitumor function by primary human macrophages in vitro, and we used this system to perform an unbiased screen of approximately 800 FDA-approved drugs. From these efforts, we identified genotype-directed targeted therapies as drugs that make *EGFR* mutant lung cancer cells more vulnerable to macrophage attack. We found these results broadly applied to lung cancers bearing other types of driver mutations and other types of targeted therapies. Our findings illuminate an innovative strategy to enhance the efficacy of targeted therapies for lung cancers with oncogenic driver mutations by combining them with anti-CD47 agents.

## Results

### Unbiased drug screens identify EGFR inhibitors as drugs that sensitize cancer cells to macrophage-mediated cytotoxicity.

**Figure 1:**
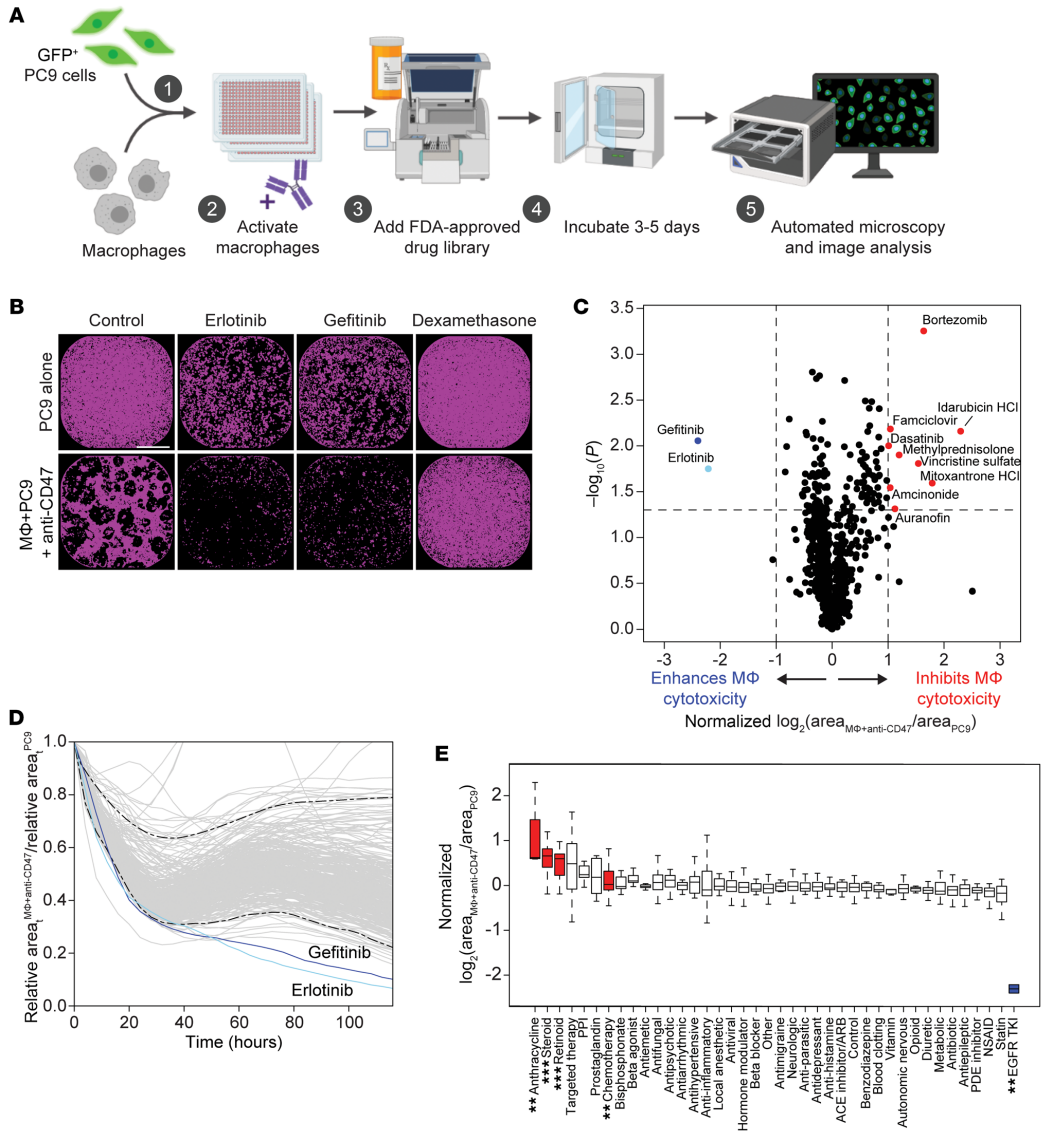
An unbiased compound library screen identifies cooperation between targeted therapy and macrophage-directed immunotherapy for *EGFR* mutant lung cancer. (**A**) Design of an unbiased functional screen to identify drugs that synergize with anti-CD47 therapy using primary human macrophages and GFP^+^ PC9 cancer cells. (**B**) Representative whole-well microscopy images showing GFP^+^ area (purple) from wells treated with drugs that enhanced (erlotinib, gefitinib) or inhibited (dexamethasone) macrophage-dependent cytotoxicity of PC9 cells. Scale bar: 800 μm. (**C**) Volcano plot summarizing drug screen results. Each point represents the mean from *n* = 5 experimental trials. The phenotypic effect size (*x* axis) is depicted as log_2_ fold change of GFP^+^ area in the macrophage+anti-CD47 condition relative to PC9 cells alone. Values were normalized to account for variation due to well position. Dashed lines represent 2-fold change in effect size (*x* axis) and *P* < 0.05 by *t* test (*y* axis). Gefitinib and erlotinib (blue) were identified as the top enhancers of macrophage-dependent cytotoxicity, whereas drugs depicted with red dots inhibited macrophage-dependent cytotoxicity or were drugs that macrophages protected against. (**D**) Curves from 1 representative plate showing macrophage-dependent cytotoxicity over time, as measured by decreases in GFP^+^ area of macrophage+anti-CD47 condition relative to the control condition. Gefitinib and erlotinib enhanced macrophage-dependent cytotoxicity within approximately 48 hours. Dashed lines indicate empirical 95% tolerance interval. (**E**) Box-and-whisker plot of drug classes ranked by normalized log_2_ fold change of GFP^+^ area in macrophage versus PC9 control condition. Boxes indicate the median and interquartile range, and whiskers indicate maxima and minima (excluding outliers) for the indicated drug class. Drug classes that significantly increased relative GFP^+^ area are depicted in red, whereas EGFR TKIs (blue) were identified as the only drug class that significantly decreased relative GFP^+^ area. Each class of drugs was compared with controls (DMSO and empty wells) using a *t* test (**FDR < 0.01, ***FDR < 0.001).

To identify drugs that make lung cancer cells more vulnerable to macrophage-mediated destruction, we developed an unbiased screening platform that measures macrophage antitumor function in a high-throughput manner (Figure 1A). We first employed this platform to study *EGFR* mutant lung cancer. We differentiated primary human macrophages ex vivo using M-CSF (CSF-1), which may polarize toward an M2-like state (19–22) and, therefore, may approximate macrophage polarization in the tumor microenvironment. We then cocultured the macrophages in 384-well plates with GFP^+^ PC9 cells, a human *EGFR* mutant lung cancer cell line (23). A small-molecule library of approximately 800 FDA-approved drugs (Supplemental Figure 1; supplemental material available online with this article; https://doi.org/10.1172/JCI169315DS1) was added to the wells at a concentration of 5.0 μM along with a CD47-blocking antibody. We then cocultured the cells for 3–5 days and performed whole-well imaging to quantify the surviving GFP^+^ area. We evaluated the ability of each drug to kill the GFP^+^ PC9 cells in the presence of activated macrophages compared with GFP^+^ PC9 cells alone. From this analysis, we identified 2 drug classes that specifically inhibit macrophage antitumor function (steroids, retinoids), and 2 drug classes that are less effective when macrophages are present (anthracyclines, other chemotherapy drugs) (Figure 1, B–E, and Supplemental Figures 2 and 3). In contrast, 2 EGFR tyrosine kinase inhibitors (TKIs) — erlotinib and gefitinib — markedly and specifically enhanced the ability of the macrophages to kill the PC9 cells. These drugs synergized with anti-CD47 therapy and resulted in over 4-fold enhancement of macrophage-mediated cytotoxicity (Figure 1, B–E, and Supplemental Figures 2–4). Given that the PC9 cells contain an activating mutation in *EGFR* (23), we hypothesized that EGFR inhibitors act on the cancer cells to prime them for macrophage-mediated destruction.

### EGFR inhibitors promote macrophage phagocytosis of EGFR mutant lung cancer cells.

**Figure 2:**
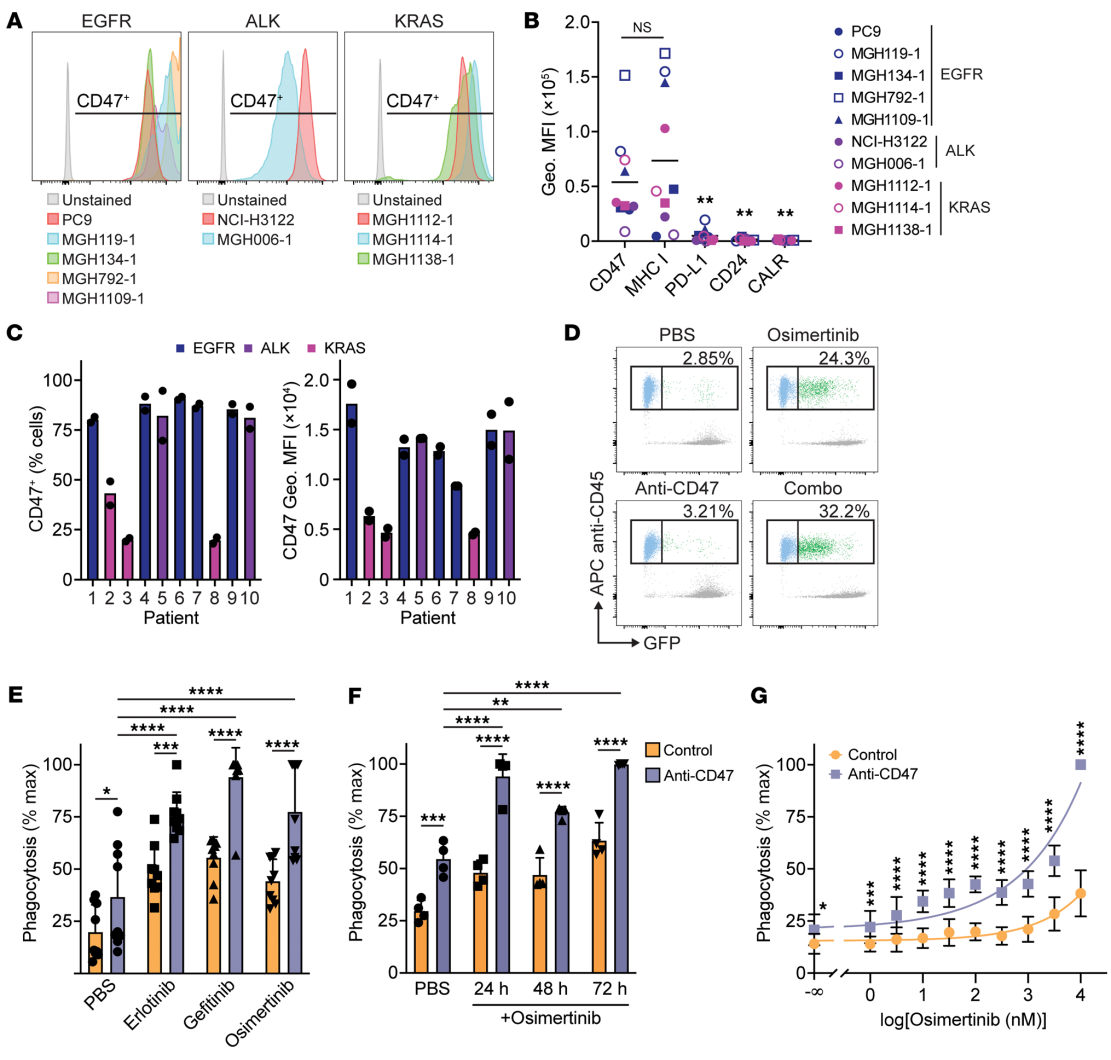
Combined targeting of EGFR and CD47 enhances macrophage phagocytosis in vitro. (**A**) CD47 expression on the surface of NSCLC cell lines containing the indicated driver mutations. (**B**) Expression of macrophage immune checkpoint molecules on the surface NSCLC cell lines containing the indicated driver mutations. Geometric mean fluorescence intensity (Geo. MFI) for each antigen was compared with CD47. (**C**) CD47 expression on EpCam^+^ cancer cells from malignant pleural effusion specimens from patients with NSCLC. Left: Percentage of CD47^+^ cells. Right: CD47 geometric MFI. Bars represent mean of 2 technical replicates (points) from *n* = 10 independent patients. (**D**) Representative analysis of phagocytosis assays by flow cytometry. PC9 cells were exposed to vehicle control (PBS) or 1 μM EGFR TKI (erlotinib, gefitinib, or osimertinib) for 24 hours and then cocultured with macrophages with or without anti-CD47 for 2 hours. Phagocytosis was measured as the percentage of macrophages (CD45^+^ cells) engulfing GFP^+^ PC9 cells. (**E**) Quantification of phagocytosis using the indicated EGFR TKIs at 1 μM concentration. Phagocytosis was normalized to the maximal response for each independent donor. Data depict mean ± SD from *n* = 9 independent blood donors combined from 3 independent experiments using CFSE^+^ or GFP^+^ PC9 cells. (**F**) Phagocytosis assays using GFP^+^ PC9 cells exposed to 1 μM osimertinib for varying amounts of time prior to coculture with macrophages (*n* = 4 independent donors). The cells were analyzed for phagocytosis as in **E**. (**G**) Phagocytosis assays using GFP^+^ PC9 cells exposed to varying concentrations of osimertinib for 24 hours prior to coculture with macrophages. Data represent mean ± SD from 2 independent experiments using a total of *n* = 8 individual donors with 3 cocultures per donor. (**B** and **E**–**G**) *P < 0.05, **P < 0.01, ***P < 0.001, ****P < 0.0001 by 1-way (**B** and **E**) or 2-way (**F** and **G**) ANOVA with Holm-Šidák multiple comparison test.

To investigate the therapeutic potential of combining EGFR inhibitors with anti-CD47 antibodies, we first examined whether CD47 could be a genuine target for lung cancers bearing driver mutations. Using flow cytometry, we evaluated cell-surface expression of CD47 on established and patient-derived cell lines containing *EGFR* or *KRAS* driver mutations or oncogenic *ALK* fusions. We found that CD47 was highly expressed on the cell surface of all specimens tested (Figure 2A). We also compared CD47 expression relative to other surface antigens that regulate macrophage activity, including MHC class I, PD-L1, CD24, and calreticulin (24–27). We found that both CD47 and MHC class I were highly expressed, whereas other macrophage immune checkpoint molecules were low or absent (Figure 2B). Both CD47 and MHC class I molecules were also highly expressed across lung adenocarcinoma specimens in the TCGA database. Expression of MHC class I genes was significantly higher for lung cancers with activating mutations in *EGFR* (Supplemental Figure 5A). In contrast, both CD47 expression and MHC class I expression was significantly lower in lung cancers with *KRAS* driver mutations (Supplemental Figure 5B). We also examined CD47 expression on primary lung cancer cells from malignant pleural effusions and observed high CD47 expression on the cell surface (Figure 2C). Together, these findings suggest expression of these molecules may vary based on the driver mutation, but both CD47 and MHC class I molecules may be important macrophage immune checkpoints for lung cancers with driver mutations.

We next determined if CD47 could exert a functional role to protect lung cancer cells from macrophage phagocytosis and whether treatment with EGFR TKIs could enhance phagocytosis. We exposed GFP^+^ PC9 cells to 1.0 μM TKI (erlotinib, gefitinib, or osimertinib) for 48 hours and then cocultured the cells with primary human macrophages for 2 hours alone or with a CD47-blocking antibody (28). Regardless of which TKI was used, maximal phagocytosis occurred with the combination of an anti-CD47 antibody and TKI-treated cells (Figure 2, D and E). This effect was maximal within 24 hours of TKI exposure (Figure 2F), a time point at which the cancer cells are actively proliferating and only exhibit minimal apoptosis (Supplemental Figure 6). Furthermore, this effect exhibited a dose-response relationship such that greater concentrations of osimertinib resulted in greater macrophage phagocytosis upon anti-CD47 treatment (Figure 2G).

### The combination of EGFR inhibitors and anti-CD47 antibodies eliminates persister cells in vitro.

**Figure 3:**
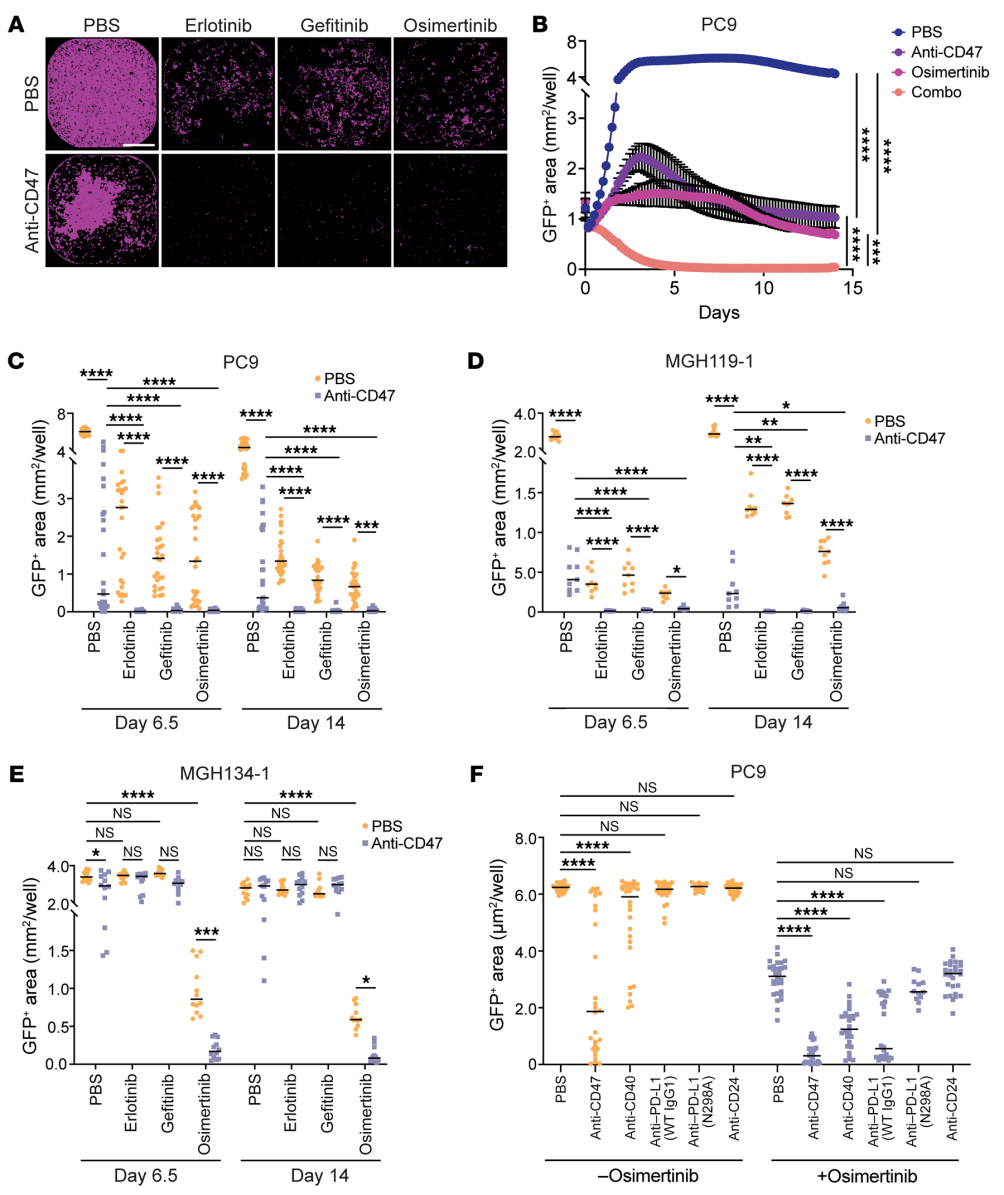
Combining TKIs with anti-CD47 antibodies eliminates *EGFR* mutant persister cells in long-term cocultures assays with primary human macrophages. GFP^+^ *EGFR* mutant lung cancer cells were cocultured with primary human macrophages and treated as indicated with vehicle control (PBS), an anti-CD47 antibody (10 μg/mL), and/or EGFR TKIs (1 μM). GFP^+^ area was measured over time as a metric of cancer cell growth or elimination. (**A**) Representative images of GFP^+^ PC9 cells on day 6.5 of coculture, with macrophages showing GFP^+^ area (purple). Scale bar: 800 μm. (**B**) Growth of GFP^+^ PC9 cells in coculture with macrophages using the indicated therapies. Data represent mean ± SEM with statistical analysis performed on day 14. (**C**) Growth of GFP^+^ PC9 cells in coculture with macrophages with or without an anti-CD47 antibody and/or EGFR TKIs as indicated. (**D**) Growth of GFP^+^ MGH119-1 patient-derived cells in coculture with macrophages with or without an anti-CD47 antibody and/or EGFR TKIs as indicated. (**E**) Growth of GFP^+^ MGH134-1 patient-derived cells in coculture with macrophages with or without an anti-CD47 antibody and/or the EGFR TKIs as indicated. MGH134-1 cells are resistant to first-generation EGFR TKIs (erlotinib, gefitinib) but sensitive to third-generation TKIs (osimertinib). (**F**) Growth of GFP^+^ PC9 cells in coculture with macrophages and the indicated macrophage immune checkpoint inhibitors (10 μg/mL). Cells were cocultured with the antibodies alone or in combination with osimertinib (100 nM). Data depict GFP^+^ area on day 6.5. (**B**–**F**) Data represent 3–4 cocultures per donor from experiments performed using a total of *n* = 3–9 independent macrophage donors. (**C**–**F**) Points represent individual cocultures, bars represent the mean. *P < 0.05, **P < 0.01, ***P < 0. 001, ****P < 0.0001 by 1-way (**B**–**E**) or 2-way (**F**) ANOVA with Holm-Šidák’s multiple comparisons test.

To model the interactions between macrophages and cancer cells as they occur over extended durations of time, we developed “long-term” coculture assays in which GFP^+^ cancer cells are cocultured with primary human macrophages and drug treatments for up to 14 days. We performed whole-well imaging over the coculture period, and the GFP^+^ area was quantified over time as a metric of cancer cell growth or death. This experimental system integrates all possible mechanisms of macrophage-mediated cytotoxicity and can evaluate persister cell formation in response to targeted therapies (29). Using these assays, we cocultured GFP^+^ PC9 cells with vehicle control, EGFR TKIs (erlotinib, gefitinib, or osimertinib), anti-CD47, or the combination of anti-CD47 and an EGFR TKI (Figure 3, A–C; Supplemental Figure 7; Supplemental Figure 8, A and B; and Supplemental Videos 1–4). At baseline, macrophages exerted no substantial antitumor effect on the PC9 cells. Each individual TKI was able to inhibit the growth of the PC9 cells by themselves, but persister cells always formed and accounted for approximately 15% of the cells after 14 days. Treatment with an anti-CD47 antibody caused the formation of patches or foci of cancer cells that remained but also was not able to fully eliminate all cancer cells from the well. However, the combination of any EGFR TKI with an anti-CD47 antibody dramatically eliminated cancer cells and prevented development of persister cells (Figure 3, A–C; Supplemental Figure 7; Supplemental Figure 8, A and B; and Supplemental Video 4). These effects were observed over a range of concentrations, with the IC_50_ of the anti-CD47 antibody improving from 223.2 ng/mL (95% CI, 158.2–317.3) to 71.25 ng/mL (95% CI, 52.39–97.22) upon combination with gefitinib (Supplemental Figure 8B). We observed similar effects using GFP^+^ MGH119-1 cells, a patient-derived *EGFR* mutant lung cancer cell line (30) (Figure 3D and Supplemental Figure 8C). Again, the combination of each TKI with an anti-CD47 antibody elicited the greatest antitumor response and eliminated or prevented the formation of persister cells.

To understand whether the effects of the combination therapy were dependent on sensitivity to a particular TKI, we also tested GFP^+^ MGH134-1 cells, a patient-derived cell line with a secondary *EGFR^T790M^* mutation (30, 31). This cell line is consequently resistant to erlotinib and gefitinib but sensitive to osimertinib. In coculture assays, only osimertinib rendered the cancer cells more vulnerable to the anti-CD47 antibody, whereas neither erlotinib or gefitinib inhibited cell growth as single agents nor in concert with CD47 blockade (Figure 3E and Supplemental Figure 8D). These findings suggest that disruption of oncogenic signaling from EGFR is required for enhanced macrophage-mediated cytotoxicity in response to anti-CD47 therapy. Furthermore, they indicate that the TKIs are primarily acting on the cancer cells rather than exerting off-target effects to the macrophages.

We next evaluated whether targeting CD47 was unique compared with other reported macrophage-directed therapies. We tested a panel of antibodies to target macrophage immune checkpoints, including CD47, CD40, PD-L1, and CD24 (25, 26, 32). Although we found that some of these molecules were not highly expressed on the cancer cells at baseline, they could be dynamically regulated in response to targeted therapies by either the cancer cells or macrophages. We found that, as single agents, only the anti-CD47 antibody and an anti-CD40 agonist antibody were able to induce significant macrophage-mediated cytotoxicity of GFP^+^ PC9 cells (Figure 3F). When combined with osimertinib, the anti-CD47 antibody elicited the greatest antitumor response and efficiently reduced persister cell numbers (Figure 3F).

### The efficacy of the combination therapy extends to lung cancers with other alterations in the RTK/MAPK pathway.

**Figure 4:**
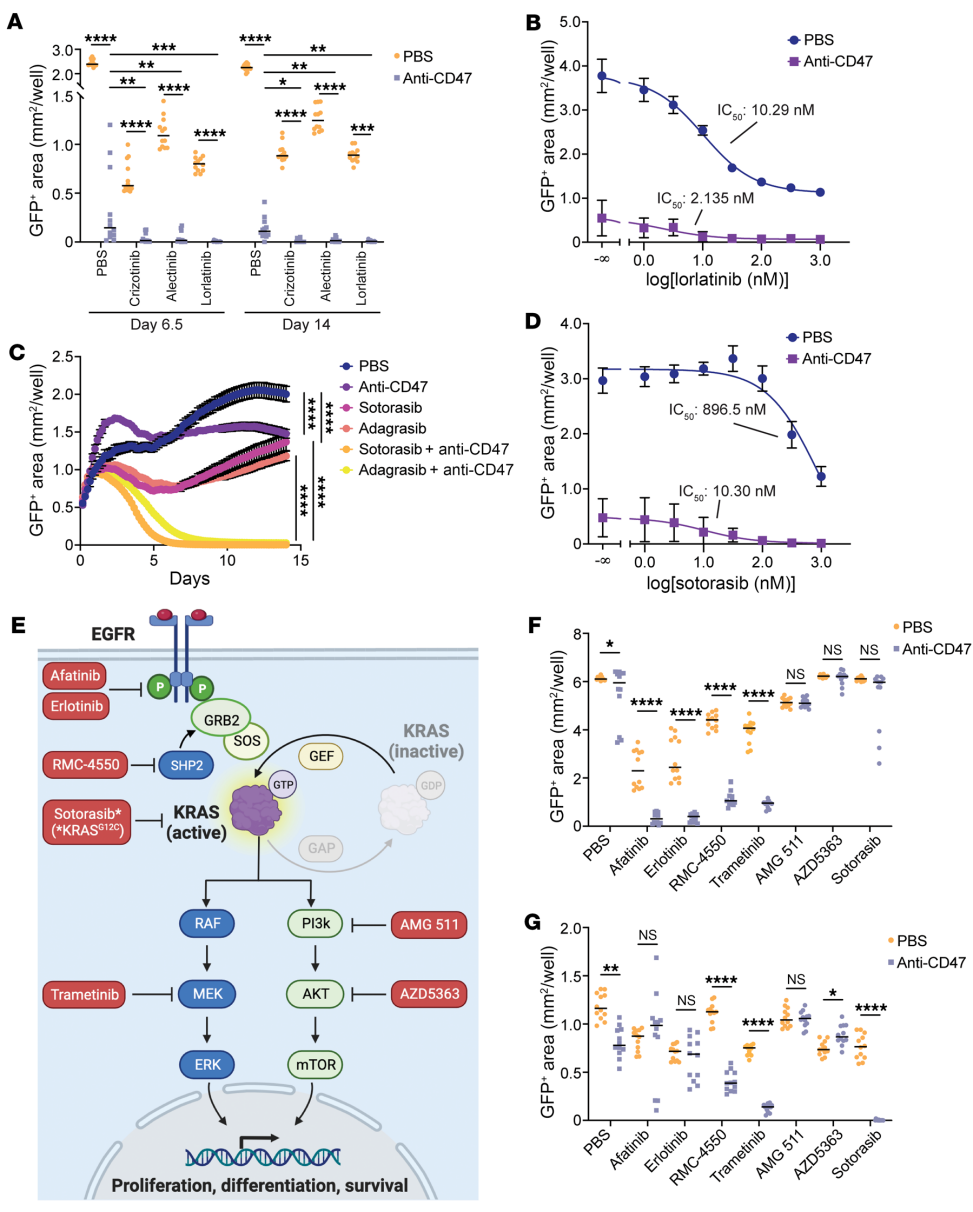
Targeted inhibition of the MAPK pathway primes NSCLC cells for macrophage-mediated destruction. (**A**) Growth of GFP^+^ NCI-H3122 cells (a human *ALK*^+^ NSCLC cell line) cocultured with primary human macrophages and treated with vehicle (PBS), an anti-CD47 antibody (10 μg/mL), and/or the indicated ALK-specific TKIs (1 μM). (**B**) Growth of GFP^+^ NCI-H3122 cells cocultured with macrophages with or without an anti-CD47 antibody (10 μg/mL) and varying concentrations of the ALK-specific TKI lorlatinib. IC_50_ of lorlatinib alone (PBS) = 10.29 nM (95% CI, 8.665–12.22) versus IC_50_ of lorlatinib+anti-CD47 = 2.135 nM (95% CI, 0.6934–6.261]). (**C**) Growth of GFP^+^ NCI-H358 cells (a human *KRAS^G12C^* mutant NSCLC cell line) cocultured with macrophages and treated with vehicle (PBS), an anti-CD47 antibody (10 μg/mL), and/or the indicated KRAS^G12C^ inhibitors (1 μM). (**D**) Growth of GFP^+^ NCI-H358 cells cocultured with macrophages with or without an anti-CD47 antibody (10 μg/mL) and varying concentrations of the KRAS^G12C^ inhibitor sotorasib. IC_50_ of sotorasib alone (PBS) = 896.5 nM (95% CI, 558.6–1,697) versus IC_50_ of sotorasib+anti-CD47 = 10.30 nM (95% CI, 2.949–40.48). (**E**) Diagram depicting the EGFR/RAS/MAPK pathway. KRAS can signal via MAPK elements or the PI3K/AKT pathway. Specific inhibitors used in this study are indicated in red. Sotorasib is specific for KRAS^G12C^. (**F**) Growth of GFP^+^ PC9 cells in coculture with macrophages with or without an anti-CD47 antibody (10 μg/mL) and varying EGFR/RAS/MAPK or PI3K/AKT pathway inhibitors. (**G**) Growth of GFP^+^ NCI-H358 cells in coculture with macrophages with or without an anti-CD47 antibody (10 μg/mL) and varying inhibitors, as in **F**. (**A**–**G**) Data represent individual cocultures with means (**A**, **F**, and **G**), mean ± SD on day 6.5 (**B** and **D**), or mean ± SEM (**C**) from 3 cocultures per donor using *n* = 4 independent macrophage donors. *P < 0.05, **P < 0.01, ***P < 0. 001, ****P < 0.0001 by 1-way ANOVA with Holm-Šidák multiple comparisons test.

Our results indicate that for *EGFR* mutant lung cancer, disabling signals from EGFR makes the cells more vulnerable to macrophage-mediated destruction. We reasoned that our findings could also apply to lung cancers containing other types of driver mutations. We therefore examined GFP^+^ NCI-H3122 cells, which contain an oncogenic *EML4-ALK* fusion (33). In coculture with macrophages, the anti-CD47 therapy significantly impaired the growth of the cancer cells as a single agent (Figure 4A and Supplemental Figure 9). ALK-specific TKIs (crizotinib, alectinib, or lorlatinib) also impaired the growth of the cancer cells as single agents, but a substantial number of persister cells remained in culture. However, the combination of an ALK inhibitor with an anti-CD47 antibody yielded the greatest antitumor response, effectively eliminating all cancer cells from the culture (Figure 4A and Supplemental Figure 9). These effects occurred with a dose-response relationship, with the IC_50_ of lorlatinib decreasing from 10.29 nM (95% CI, 8.665–12.22) to 2.135 nM (95% CI, 0.6934–6.261) with the combination of an anti-CD47 antibody (Figure 4B).

Similarly, we investigated cell lines with mutations in *KRAS*, one of the most commonly mutated genes in lung cancer (1). We performed coculture assays using macrophages and NCI-H358 cells, a human lung cancer cell line containing a *KRAS^G12C^* activating mutation (34). In long-term coculture assays, we found that anti-CD47 antibodies or KRAS^G12C^ inhibitors (sotorasib or adagrasib) inhibited cancer cell growth over time but generally had only moderate effects as single agents (Figure 4C). In contrast, the combination of the two therapies had a striking effect, with dramatic elimination of tumor cells from culture (Figure 4C). As above, we performed titrations of sotorasib and determined that a dose-response relationship existed and that combination therapy substantially decreased the IC_50_ of sotorasib (896.5 nM, 95% CI, 558.6–1697 nM; versus 10.30 nM, 95% CI, 2.949–40.48 nM) (Figure 4D).

To rigorously evaluate the effects of the combination therapy on persister cells, we performed coculture assays with varying numbers of cancer cells followed by drug washout. We cocultured the cells with macrophages for 14 days in the presence of an anti-CD47 antibody, appropriate targeted therapies, or the combination. We then washed out the drugs, added fresh medium, and evaluated cancer cell regrowth after an additional 9 days of culture. For nearly all conditions tested, we found that treatment with the combination therapy prevented or reduced cancer cell regrowth, indicating there were fewer persister cells at the time of washout (Supplemental Figure 10, A–D). This effect was cancer cell intrinsic, because A549 cells, an NSCLC cell line that lacks an actionable driver mutation, did not exhibit similar enhancement as a result of the combination therapy (Supplemental Figure 10E).

Furthermore, to formally demonstrate that true synergy occurs between the anti-CD47 antibody and targeted therapies, we performed coculture assays using a dose-titration matrix to test 64 different dose-combinations over time. Using MuSyC analysis (35), we found that synergy could occur with respect to maximal efficacy, potency, and/or cooperativity for the combination of an anti-CD47 antibody with EGFR TKIs, lorlatinib, or sotorasib when used with their respective cancers (Supplemental Figure 11 and Supplemental Table 1). We also found that synergy could occur when using osimertinib in combination with an anti-CD40 antibody or an anti–PD-L1 antibody with a functional Fc domain, again suggesting the effects of the combination therapy may extend more broadly to other macrophage-directed therapies (Supplemental Table 2).

Since EGFR and other receptor tyrosine kinases (RTKs) can transduce growth signals via the MAPK pathway and/or the PI3K/AKT pathway, we tested a panel of inhibitors to dissect the molecular mediators underlying the effects of the combination therapy (Figure 4E). Using coculture assays with *EGFR* mutant PC9 cells, we found that any active inhibitor of the MAPK pathway could be enhanced by combination with an anti-CD47 antibody, whereas no significant enhancement was observed when combining an anti-CD47 antibody with AKT or PI3K inhibitors (Figure 4F). Similarly, using *KRAS^G12C^* mutant NCI-H358 cells, we found that inhibition of SHP-2, KRAS^G12C^, or MEK could be enhanced by combining with anti-CD47 therapy, whereas no significant enhancement was observed when combining with PI3K or AKT inhibitors (Figure 4G). These findings suggest that efficacy of the combination therapy is specifically dependent on inhibition of the MAPK pathway rather than alternative signaling pathways.

To understand changes in macrophage activation states as a consequence of the combination therapy, we performed multiparameter flow cytometry, multiplex cytokine analysis, and transcriptional profiling. In coculture assays, macrophages exhibited increased phagocytosis in response to either targeted therapies or anti-CD47 antibodies as single agents (Supplemental Figure 12). Treatment with the combination therapy elicited upregulation of the M1 markers CD86 and MHC II with relative downregulation of the M2 markers CD163 and CD206 (Supplemental Figure 13). We also found that the combination of targeted therapies and anti-CD47 could elicit secretion of proinflammatory cytokines and chemokines, including MIP-1α, MIP-1β, MIP1Δ, RANTES, MCP-2, and MCP-4 (Supplemental Figure 14, A and B). For some cytokines, such as MIP-1α, we observed direct secretion by macrophages exposed to the combination therapy even in the absence of cancer cells. Transcriptional profiling identified 10 genes that were specifically upregulated in macrophages that had been cocultured with cancer cells and treated with the combination therapy. This gene set included cytokines, such as *CXCL3* and *CXCL5*, and phagocytic receptors, such as *FCGR3A* and *MARCO* (Supplemental Figure 14C). These profiling experiments emphasize the robust antitumor state of the macrophages in response to the combination treatment.

### The combination of targeted therapies and CD47 blockade is effective in mouse tumor models bearing driver mutations.

**Figure 5:**
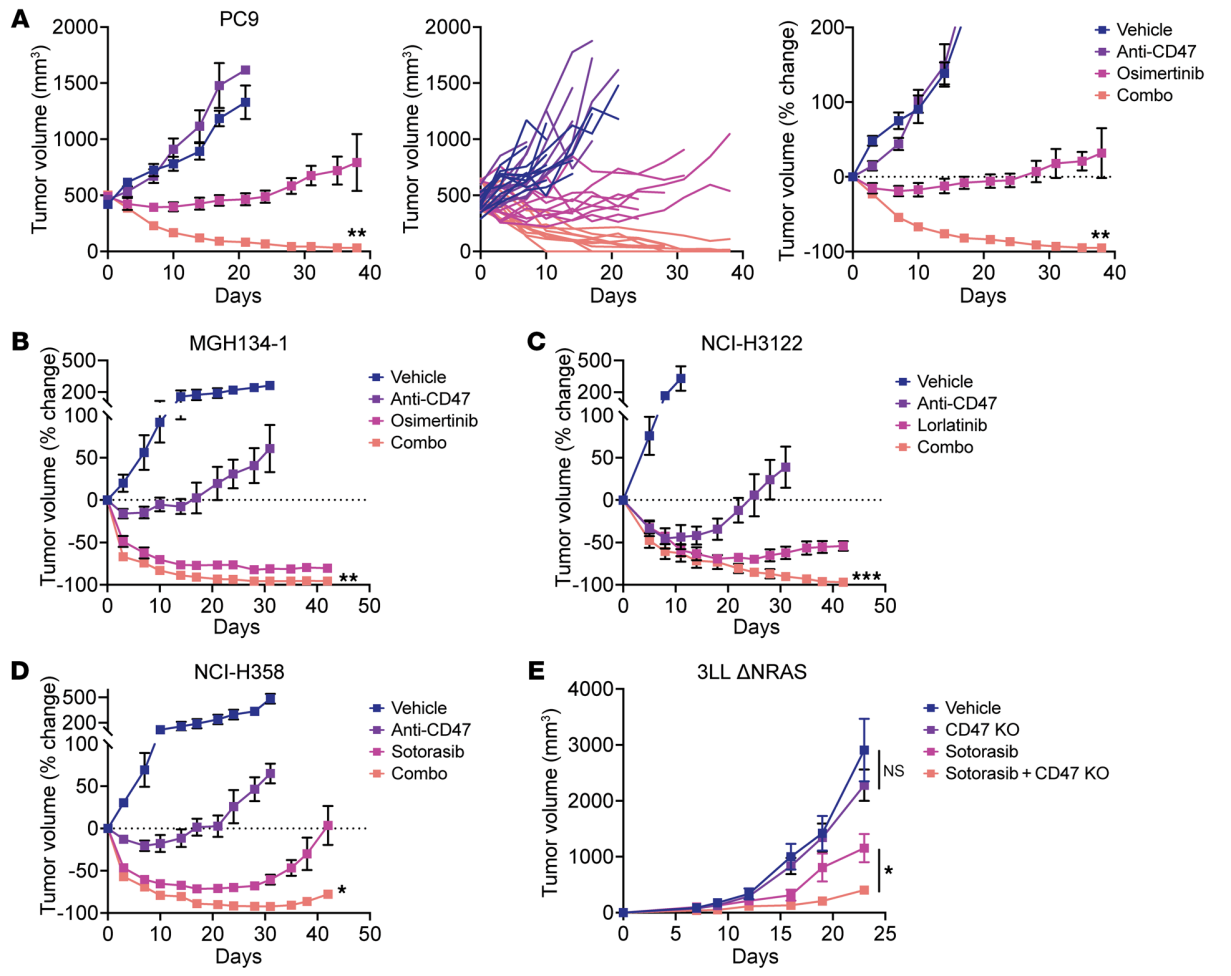
The combination of targeted therapy and CD47 blockade enhances antitumor responses in mouse tumor models. (**A**) *EGFR* mutant NSCLC PC9 xenograft model using NSG mice. Tumors were grown to approximately 500 mm^3^ and then mice were treated with vehicle control, an anti-CD47 antibody (250 μg 3 times weekly), osimertinib (5 mg/kg 5 times weekly), or the combination of anti-CD47 and osimertinib. Data depict mean tumor volume ± SEM (left), growth curves from individual mice (middle), or change in tumor volume from baseline (right). Complete responses were observed in 4 of 10 mice (40%) in the combination cohort. Data represent *n* = 9–11 mice per cohort combined from 2 independent experiments. (**B**) *EGFR* mutant NSCLC xenograft model of MGH134-1 patient-derived cells engrafted into NSG mice and treated as in **A**. (**C**) *ALK*^+^ xenograft model of NCI-H3122 cells engrafted into NSG mice and treated with vehicle control, an anti-CD47 antibody (250 μg 3 times weekly), lorlatinib (6 mg/kg 5 times weekly), or the combination of anti-CD47 and lorlatinib. (**D**) *KRAS^G12C^* mutant xenograft model of NCI-H358 cells engrafted into NSG mice and treated with vehicle control, an anti-CD47 antibody (250 μg 3 times weekly), sotorasib (100 mg/kg 5 times weekly), or the combination of anti-CD47 and sotorasib. (**E**) Syngeneic model of *KRAS^G12C^* mutant lung cancer using wild-type 3LL ΔNRAS cells or a CD47-KO variant engrafted into C57BL/6 mice. The mice were treated with vehicle control or sotorasib (30 mg/kg 5 times weekly) starting day 7 after engraftment. Data represent mean ± SEM from *n* = 9–10 mice per cohort. *P < 0.05 by paired *t* test for the indicated comparisons. (**B**–**D**) Data represent change in tumor volume from baseline with mean ± SEM of *n* = 4 mice per cohort. (**A**–**D**) *P < 0.05, **P < 0.01, ***P < 0. 001 by unpaired *t* test for combination versus targeted therapy.

To study the efficacy of the combination therapy in vivo, we first employed xenograft models of human lung cancer. We used NSG mice, which lack functional T, B and NK cells but contain macrophages that can be stimulated to attack tumors (16, 28, 36, 37). Importantly, NSG mice have an allele of SIRPa that cross-reacts with human CD47; therefore, they have been used as a gold-standard model for evaluating CD47-blocking therapies in vivo (16, 28, 37, 38). We engrafted mice subcutaneously with PC9 cells and allowed tumors to grow to approximately 500 mm^3^. Mice were then randomized to treatment with vehicle control, an anti-CD47 antibody, osimertinib, or the combination of osimertinib and the anti-CD47 antibody (Figure 5A). As a single agent, the anti-CD47 antibody produced no significant inhibition of tumor growth. Treatment with osimertinib as a single agent was able to inhibit tumor growth, but tumors gradually progressed over time. Remarkably, treatment with the combination therapy dramatically reduced tumor burden and elicited complete elimination of tumors in several animals (Figure 5A). We also tested a patient-derived xenograft model of *EGFR* mutant lung cancer (MGH134-1) and again observed the greatest antitumor effects from the combination treatment of osimertinib with an anti-CD47 antibody (Figure 5B and Supplemental Figure 15).

To understand whether these findings could extend to other types of lung cancer bearing different driver mutations, we tested models of *ALK*^+^ lung cancer (NCI-H3122 cells) and *KRAS^G12C^* mutant lung cancer (NCI-H358 cells). In each of these models, the greatest antitumor effects were observed with the combination of targeted therapy (lorlatinib for NCI-H3122 cells, sotorasib for NCI-H358 cells) and an anti-CD47 antibody (Figure 5, C and D, and Supplemental Figure 15), consistent with our observations in vitro. Similarly, we tested an immunocompetent, syngeneic model of *KRAS^G12C^* mutant lung cancer using 3LL ΔNRAS cells, a variant of Lewis lung carcinoma that harbors an endogenous *KRAS^G12C^* mutation and responds to KRAS inhibitors (39). In this model, we found that genetic ablation of CD47 had no significant effect on tumor growth by itself (Figure 5E and Supplemental Figure 16). Sotorasib was able to inhibit tumor growth as a single agent, yet the greatest inhibition of tumor growth occurred upon sotorasib treatment of a CD47-KO cell line (Figure 5E). Together, these findings indicate that our in vitro findings translate to in vivo models and that dual blockade of CD47 and oncogenic drivers can enhance antitumor responses to lung cancer.

### β2-Microglobulin and CD73 are “don’t-eat-me” signals that can be altered by targeted therapies.

**Figure 6:**
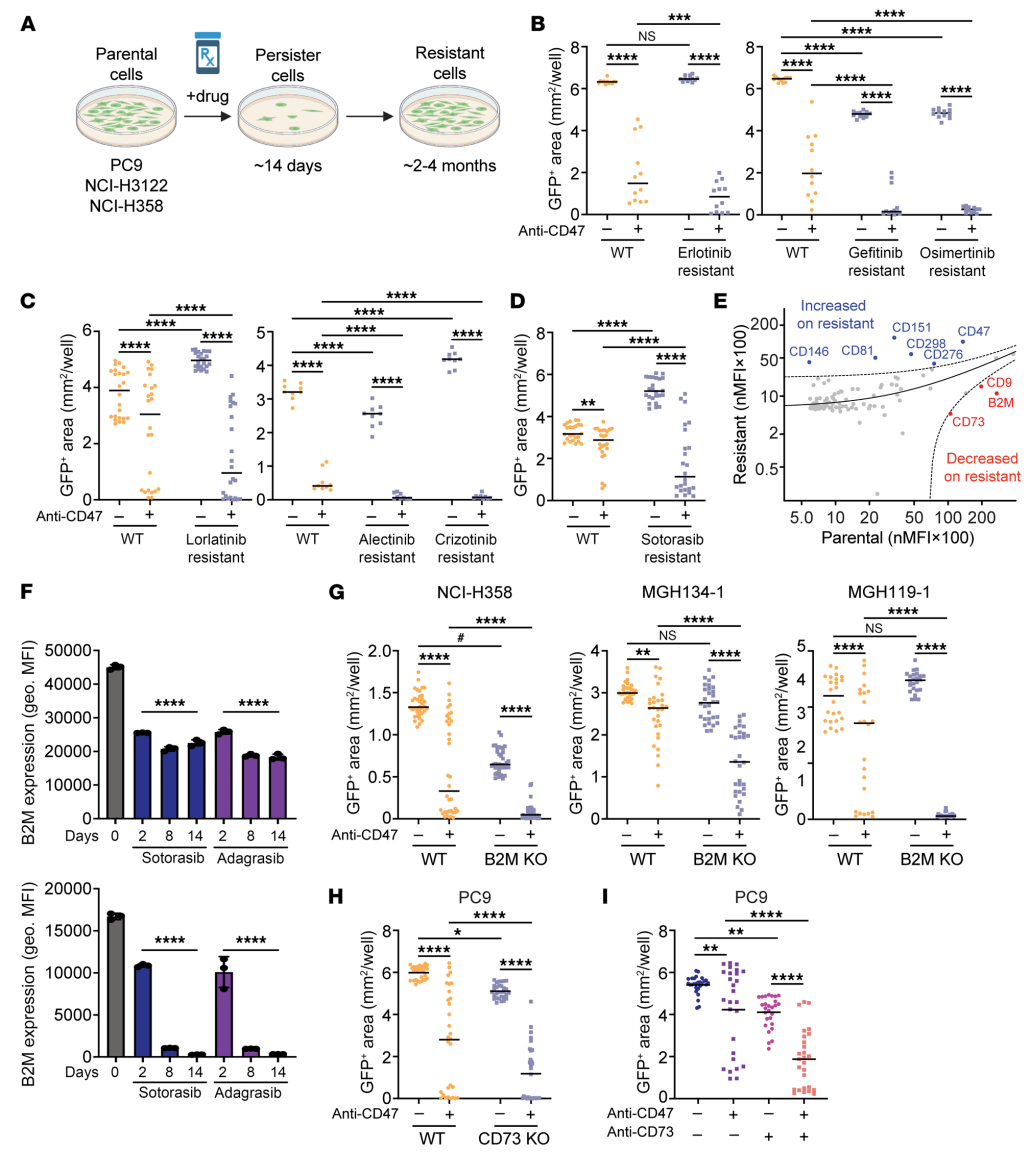
Targeted therapies induce cross-sensitization to anti-CD47 therapy and downregulate B2M and CD73. (**A**) Generation of GFP^+^ cell lines that are resistant to targeted therapies. Cells were cultured with 1.0 μM of appropriate targeted therapy until resistant cells emerged and proliferated in culture. (**B**–**D**) Long-term coculture assays using GFP^+^ PC9 cells (**B**), GFP^+^ NCI-H3122 cells (**C**), or GFP^+^ NCI-H358 cells (**D**) that are resistant to the indicated targeted therapies. Anti-CD47 therapy significantly enhanced macrophage-mediated cytotoxicity of each resistant line relative to its naive parental counterpart. (**E**) Scatter plot showing results of comprehensive surface immunophenotyping of parental NCI-H358 cells versus a GFP^+^ sotorasib-resistant variant. Each dot represents the normalized mean fluorescence intensity (nMFI) of an individual surface antigen from a total of 354 specificities tested in 1 experiment. Antigens that exceed the 95% predicted interval on the parental line (red) or resistant line (blue) are indicated. (**F**) Treatment of parental NCI-H358 cells with the indicated targeted therapies causes downregulation of B2M (top) and CD73 (bottom) over time as measured by flow cytometry. ****P < 0.0001 for each drug treatment condition compared with time = 0 hours by 1-way ANOVA with Tukey’s multiple comparison test. (**G**) Evaluation of wild-type versus B2M KO lung cancer cell lines in long-term coculture assays with human macrophages. (**H**) Evaluation of wild-type versus CD73 KO PC9 cells in long-term coculture assays with human macrophages. (**I**) Treatment of PC9 cells with a CD73-blocking antibody alone or in combination with anti-CD47 in coculture assays with human macrophages. (**B**–**D** and **G**–**I**) Data represent experiments performed with *n* = 4–12 independent macrophage donors. Points represent individual cocultures, bars represent means at 6.5 days. *P < 0.05, **P < 0.001, ***P < 0.001, ****P < 0.0001 by 2-way ANOVA with Holm-Šidák multiple comparisons test; ^#^GFP^+^ area was underrepresented due to high confluency and was not visually different by phase microscopy.

To understand the mechanisms by which targeted therapies make cancer cells more vulnerable to macrophage-directed therapies, we generated a panel of 7 GFP^+^ lung cancer cell lines that are each resistant to their respective targeted therapies. The cell lines were generated by prolonged culture in 1 μM of appropriate targeted therapy until resistant cells emerged and grew at rates comparable to their naive parental counterparts (Figure 6A and Supplemental Figure 17). The lines included PC9 cells (resistant to erlotinib, gefitinib, or osimertinib), NCI-H3122 cells (resistant to crizotinib, alectinib, or lorlatinib), and NCI-H358 cells (resistant to sotorasib). As a consequence of becoming drug resistant, we found that each cell line also became more sensitive to macrophage-mediated cytotoxicity in response to anti-CD47 therapy (Figure 6, B–D). We hypothesized that changes in cell-surface proteins likely mediated this effect, since these proteins are required for intercellular interactions between macrophages and cancer cells. Therefore, we performed comprehensive surface immunophenotyping of naive parental versus sotorasib-resistant NCI-H358 cell lines to identify differentially expressed surface antigens (Figure 6E). We found two known immunoinhibitory factors, β2-microglobulin (B2M) and CD73 (24, 40), that were substantially downregulated on the sotorasib-resistant line. Moreover, we found that B2M and CD73 were significantly downregulated on additional resistant cell lines and could be downregulated as a consequence of initial treatment with targeted therapies (Figure 6F and Supplemental Figure 18). To evaluate the functional contributions of these surface proteins, we generated KO cell lines using CRISPR/Cas9 (Supplemental Figure 19). B2M KO abrogated expression of MHC class I molecules on the cell surface as expected (41) (Supplemental Figure 19). At the functional level, we found that genetic deletion of B2M could influence macrophage killing in response to anti-CD47 therapy for the majority of cell lines tested (Figure 6G and Supplemental Figure 20). In contrast, CD73 KO seemed to act as a “don’t-eat-me” signal only for PC9 cells, and a CD73-blocking antibody was also effective in this setting (Figure 6, H and I, and Supplemental Figure 20). These findings indicate both B2M and CD73 can act as functional macrophage immune checkpoints for lung cancers with driver mutations and that their downregulation can contribute to vulnerability to macrophage attack. Importantly, individual cancer specimens may differentially rely on these distinct immune checkpoints to evade macrophage-mediated cytotoxicity.

## Discussion

To identify therapies that sensitize cancer cells to macrophage-mediated destruction, we performed an unbiased, cell-based functional screen of approximately 800 FDA-approved drugs using primary human macrophages as effectors. We identified genotype-directed targeted therapies as those that prime lung cancer cells for macrophage-mediated destruction. In subsequent in vitro and in vivo validation studies, we found these results extended to multiple NSCLC models harboring diverse oncogenic driver alterations treated with their corresponding targeted therapies. Notably, our findings complement and advance those from a recent study by Hu and colleagues who found that KRAS^G12C^ inhibitors could enhance innate immune responses and recruit more macrophages to the tumor microenvironment (42). Our findings may generalize more broadly to other macrophage-activating therapies such as anti-CD40 antibodies. Interestingly, the effects of the combination therapy may be unique to inhibition of the RTK/MAPK pathway, as similar enhancement was not observed as a class effect of chemotherapy drugs in our screen or when using AKT or PI3K inhibitors. Our findings have immediate translational implications since they suggest the combination of targeted therapies and CD47-blocking therapies could be an optimal strategy for treating patients with lung cancers with driver mutations. Furthermore, we also demonstrate that cell-intrinsic resistance to targeted therapies can cross-sensitize to CD47 blockade.

To date, clinical trials combining targeted therapies and T cell–directed immune checkpoint inhibitors have not been successful for lung cancer. These studies have either been limited in efficacy or demonstrated excessive toxicity. As an example, the combination of osimertinib with an anti–PD-L1 antibody showed severe side effects in nearly 50% of patients (8). Macrophage-directed therapies are an orthogonal treatment modality and may benefit different patients than T cell–directed therapies. This is particularly true since macrophages have inherent ability to kill cancer cells when provided with an appropriate stimulus, whereas T cell cytotoxicity is intertwined with the tumor mutational burden and the presence of neoantigens. Interestingly, we found that downregulation of B2M and CD73 could contribute to enhanced sensitivity to macrophage killing. B2M is required for MHC class I expression on the cell surface, which CD8 T cells depend on for antigen recognition. However, B2M also acts as a don’t-eat-me signal by binding to LILRB1, an inhibitory receptor on macrophages (24). Downregulation of B2M could decrease antigen presentation to make cancer cells resistant to T cell–directed immunotherapies while simultaneously making them more vulnerable to macrophage attack. Thus, B2M expression may reflect a critical pivot point between innate and adaptive immune activation. Similarly, CD73 is an ectoenzyme that catalyzes the breakdown of AMP to immunosuppressive adenosine in the tumor microenvironment (40). Its downregulation may make lung cancer cells more sensitive to macrophage-mediated cytotoxicity. Our findings suggest lung cancer specimens may differentially rely on these immunosuppressive signals to evade detection by macrophages.

The high-throughput screening platform we developed is a robust system to identify drugs that activate or inhibit macrophage-mediated cytotoxicity. Of note, our screen also identified putative drug-drug interactions that may negatively affect CD47-blocking therapies or other macrophage-directed therapies for cancer. For example, steroids and retinoids were classes of drugs that were generally inhibitory to macrophages and abrogated the effects of anti-CD47 therapy. Moreover, anthracyclines were effective at killing cancer cells by themselves as single agents, but they were completely inactive in the presence of macrophages. These findings warrant further investigation in vivo, but they suggest these drug classes should be limited or avoided when treating patients with macrophage-directed therapies. In the future, our screening platform could be applied to high-throughput investigation of larger compound libraries. Since the platform uses functional interrogation of primary human macrophages, it is target agnostic and therefore offers great opportunity to understand fundamental biology and discover new drug candidates.

As limitations of our study, our experimental approach focused on in vitro studies using primary human macrophages, xenograft mouse models, and syngeneic immunocompetent mouse models. Although the combination of these studies has been useful to predict the safety and efficacy of CD47-blocking therapies in clinical trials, all preclinical models have inherent limitations, and it is possible that our findings do not translate to patients as expected. As another limitation, toxicity has been observed with some anti-CD47 agents, primarily relating to on-target toxicity to normal, healthy red blood cells that express CD47 on their surface (15, 43, 44). Targeted therapies for lung cancer generally do not have substantial effects on red blood cells (2, 3, 5); therefore, we do not expect enhanced toxicity from the combination strategy. However, this possibility should be evaluated and monitored further in clinical trials. Finally, our study focused exclusively on lung cancers with driver mutations. We expect our findings will extend to other types of cancers, particularly those with RTK/MAPK pathway mutations, but further experimentation is needed to investigate these principles.

Further studies will be useful to define biomarkers that predict responses to anti-CD47 therapies. We found that expression of CD47 and MHC class I could vary in lung adenocarcinoma specimens depending on their driver mutation. For example, *EGFR* mutant lung cancers may express higher levels of MHC I molecules, while *KRAS* mutant cancers may express lower levels of CD47 and MHC I molecules. The functional consequences of these differences are uncertain at this time since both types of cancer responded to the combination therapy in vitro and in vivo. It will be important to determine if differences in expression of these molecules predict which patients may respond best to targeted therapies or macrophage-directed therapies in the clinic.

In order to translate our discoveries to patients, investigation of the combination strategy in clinical trials is warranted. There exist multiple FDA-approved targeted therapies for lung cancer (4), and multiple CD47-blocking therapies are now under investigation in clinical trials (45). Thus, combining these two treatment strategies is feasible and can occur in the near term. Our data suggest the most effective setting to test the combination strategy would be in patients with lung cancers with oncogenic driver mutations that are naive to targeted therapy. However, our data also suggest CD47-blocking therapies may be effective as single agents in patients who have progressed on targeted therapy. Overall, our findings indicate that combining targeted therapies with CD47-blocking agents or other macrophage-directed therapies may be an ideal way to merge the fields of precision medicine and immuno-oncology for the benefit of patients.

## Methods

### Sex as a biological variable.

This study includes analysis of primary human immune cells and human cancer cells. Since the immune cells in this study derived from anonymized human blood samples, we expect that both male and female samples are included in our analysis, but demographic information was not available for us to assess sex as a biological variable. Our animal experiments were performed using age- and sex-matched mice, but sex was not specifically tested as a biological variable.

### Cell lines.

PC9, NCI-H3122, and NCI-H358 cells were obtained from the MGH Center for Molecular Therapeutics (Massachusetts General Hospital). Cell line identities were confirmed by STR profiling. Mycoplasma testing was performed prior to banking frozen stocks used in this study and when any uncertainty in culture conditions arose. Patient-derived cell lines (MGH119-1, ref. 30; MGH134-1, ref. 31; MGH792-1–*EGFR* mutant NSCLC; MGH006-1–*ALK*^+^ NSCLC; MGH1112-1; MGH1114-1; MGH1138-1–*KRAS^G12C^* NSCLC) were generated using previously described methods (31). Prior to cell line generation, the patients signed informed consent to participate in a Dana Farber/Harvard Cancer Center Institutional Review Board–approved protocol giving permission for research to be performed on their sample. Human NSCLC cell lines were cultured in RPMI (Thermo Fisher Scientific) supplemented with 10% ultra-low IgG fetal bovine serum (Thermo Fisher Scientific), 100 units/mL penicillin, 100 μg/mL streptomycin, and 292 μg/mL L-glutamine (Thermo Fisher Scientific). 3LL ΔNRAS cells were provided by the lab of Julian Downward (The Francis Crick Institute, London, United Kingdom) and were cultured in DMEM (Thermo Fisher Scientific) supplemented with 10% ultra-low IgG fetal bovine serum, 100 units/mL penicillin, 100 μg/mL streptomycin, and 292 μg/mL L-glutamine. Cell lines were maintained in humidified incubators at 37°C with 5% carbon dioxide.

GFP^+^ lines were generated by lentiviral transduction of cell lines using CMV-GFP-T2A-Luciferase prepackaged virus (Systems Bio). Transduced cells were then sorted for stable GFP expression.

### Genetic modification of cell lines.

KO cell lines were generated by CRISPR/Cas9-mediated genome editing. A CD47-KO variant of 3LL ΔNRAS cells was generated using Gene Knockout Kit version 2 targeting murine CD47 (Synthego). Knockout variants of PC9, NCI-H358, MGH119, and/or MGH134 were generated using Gene Knockout Kit version 2 targeting human B2M or human CD73 (Synthego). Gene KO was performed via ribonucleoprotein transfection with recombinant Cas9 (Synthego). Cells were then stained for surface antigen expression and sorted using a FACSAria II (BD Biosciences) to generate negative cell lines. For murine CD47, staining was performed using APC anti-murine CD47 clone miap301 (BioLegend) and sorting was used to generate a clonal population. For human lines, staining was performed with APC anti-human B2M clone 2M2 (Biolegend) or APC anti-human CD73 clone AD2 (Biolegend) and used to sort polyclonal lines that were negative for surface antigen expression.

### Ex vivo generation of primary human macrophages.

Primary human macrophages were generated from peripheral blood mononuclear cells as previously described (16). Briefly, leukocyte reduction chambers were obtained from healthy human donors from discarded apheresis products via the Crimson Core Biobank. Monocytes were labeled using StraightFrom Whole Blood CD14 MicroBeads (Miltenyi Biotec) and purified using an autoMACS Pro Separator (Miltenyi Biotec). Monocytes were then cultured in IMDM (Thermo Fisher Scientific) supplemented with 10% ultra-low IgG fetal bovine serum, 100 units/mL penicillin, 100 μg/mL streptomycin, 292 μg/mL L-glutamine, and 20 ng/mL human M-CSF (Peprotech) for at least 7 days. Cells were passaged or replated as necessary and typically maintained in culture for 2–4 weeks.

### FDA drug library screen.

GFP^+^ PC9 cells and primary human macrophages were cocultured in 384-well plates in IMDM supplemented with 10% ultra-low IgG fetal bovine serum, 100 units/mL penicillin, 100 μg/mL streptomycin, 292 μg/mL L-glutamine, and 20 ng/mL human M-CSF. Purified anti-CD47 antibody clone B6H12 (BioXCell or eBioscience) was added at a working concentration of 10 μg/mL. Duplicate control plates were plated with GFP^+^ PC9 cells alone. A curated library of approximately 800 FDA-approved drugs was transferred to the plates via Echo Acoustic Liquid Handler (Koch Institute High-Throughput Sciences Facility) for a final concentration of 5 μM. Cells were then incubated for 3–5 days. Wells were imaged using automated fluorescence microscopy with an Incucyte S3 system (Sartorius). Automated image analysis was performed using Incucyte Analysis Software (Sartorius) to quantify the percentage of GFP^+^ area occupied by target cells in each well. To assess macrophage-dependent cytotoxicity, GFP^+^ area in experimental wells (containing GFP^+^ PC9 cells, macrophages, and anti-CD47 antibodies) was normalized to GFP^+^ area in control wells (containing GFP^+^ PC9 cells alone). Library screens were independently repeated *n* = 5 times, each time using macrophages derived from individual donors. For individual compounds, *P* values were computed using a 1-sample *t* test after controlling for row, column, and plate effects (46).

### FACS analysis.

Analysis of cell surface antigens was performed using live cells in suspension. Cells were blocked with 100 μg/mL mouse IgG (Lampire Biological Laboratories) or Human TruStain FcX (BioLegend). The following antibodies were used for FACS analysis: APC-conjugated anti-CD47 clone B6H12 (Thermo Fisher Scientific); Alexa Fluor 647–conjugated anti-HLA-A,B,C (MHC class I) clone W6/32 (BioLegend); APC-conjugated anti–PD-L1 clone 29E.2A3 (BioLegend); Alexa Fluor 647–conjugated anti-CD24 clone ML5 (BioLegend); PE-conjugated anti-CALR clone FMC 75 (Abcam); APC anti-CD45 clone 2D1 or clone HI30 (BioLegend); APC anti-B2M clone 2M2 (BioLegend); and APC anti-CD73 clone 2A2 (BioLegend). Viability of cell lines was assessed by staining with 100 ng/mL DAPI (MilliporeSigma). For analysis of primary pleural fluid specimens, Alexa Fluor 488–conjugated anti-EpCam clone 9C4 (BioLegend) was used to mark the malignant cell population. Cells were analyzed using an LSR Fortessa equipped with a High Throughput Sampler (BD Biosciences). Comprehensive surface immunophenotyping was performed using LEGENDScreen Human PE Kit containing 371 pretitrated antibodies and 354 unique specificities (BioLegend). The experimental run was multiplexed using GFP^+^ NCI-H358 cells resistant to sotorasib combined with nonfluorescent, parental NCI-H358 cells. Compensation was performed prior to analysis on an LSR Fortessa using FACSDiva software (BD Biosciences).

### Targeted therapies.

The following targeted therapies were used in this study: erlotinib, gefitinib, osimertinib, crizotinib, alectinib, lorlatinib, sotorasib, and adagrasib (Selleckchem). MAPK pathway inhibitors included afatinib, erlotinib, RMC-4550, sotorasib, trametinib, AMG 511, and AZD5363 (Selleckchem).

### Phagocytosis assays.

In vitro phagocytosis assays were performed as previously described (16). Briefly, GFP^+^ PC9 cells or CFSE^+^ PC9 cells were used as target cells. Labeling with CFSE (Thermo Fisher Scientific) was performed according to manufacturer’s instructions. Target cells were exposed to EGFR TKIs (erlotinib, gefitinib, or osimertinib) for 24–72 hours. Live, adherent cells were then collected, washed, and cocultured with primary human macrophages at a target/macrophage ratio of 4:1. Cells were cocultured in the presence or absence of 10 μg/mL purified anti-CD47 antibody clone B6H12. Cells were cocultured for 2 hours in serum-free IMDM in round-bottom ultra-low attachment 96-well plates (Corning). After the incubation period, cells were washed and analyzed by flow cytometry. Macrophages were identified using APC anti-CD45 clone 2D1 or clone HI30, and target cells were identified by CFSE or GFP fluorescence. Phagocytosis was quantified as the percentage of macrophages that contained CFSE or GFP^+^ signal. Phagocytosis was normalized to the maximal response by each independent macrophage donor. Dose-response curves were generated using Prism version 9.2.0 (GraphPad).

### Long-term coculture assays.

Long-term coculture of primary human macrophages was performed using GFP^+^ target cells. Cells were cultured in phenol-red free IMDM (Thermo Fisher Scientific) supplemented with 10% ultra-low IgG fetal bovine serum, 100 units/mL penicillin, 100 μg/mL streptomycin, 292 μg/mL L-glutamine, and 20 ng/mL human M-CSF. Purified anti-CD47 antibody clone B6H12 was added at a working concentration of 10 μg/mL. Targeted therapies were added at a final working concentration of 1.0 μM or as indicated otherwise. Cells were then cocultured for 7–14 days, with whole-well imaging of phase contrast and GFP channels performed every 4 hours using an Incucyte S3 system. Automated imaging analysis was performed using Incucyte Analysis Software. Images taken on day 6.5 were used as a standard reference time point for data comparison and statistical analysis. For dose-response experiments, sigmoidal dose-response curves were generated using Prism version 9.2.0 (GraphPad) to determine IC_50_ parameters. To incorporate biological variation across different donors, differentiation conditions, and coculture specimens, the sample size for statistical analysis included independent cocultures deriving from 4 to 12 macrophage donors or as otherwise indicated.

### Macrophage immune checkpoint experiments.

In long-term assays to assess macrophage immune checkpoint targeting, the following macrophage-directed therapeutics were used: purified anti-CD47 clone B6H12, anti-CD40 clone G28.5 (BioXCell), anti-hPD-L1-hIgG1 (Invivogen), anti-hPD-L1-hIgG1 (N298A) (Invivogen), purified anti-CD24 clone SN3 (GeneTex), and purified anti-CD73 clone AD2 (BioLegend).

### In vivo treatment models.

NOD.Cg-Prkdc^scid^ Il2rg^tm1Wjl^/SzJ (NSG) mice (The Jackson Laboratory) were used for all xenograft tumor models. Cells were implanted subcutaneously into the flank of NSG mice (6–8 weeks of age) in 0.2 mL 50% BD Matrigel Basement Membrane Matrix in PBS. Tumor volumes were measured twice weekly using electronic calipers and calculated using the formula: mm^3^ = 0.52 × *L* × *W*^2^. Mice were randomized to treatment cohorts when tumor volumes reached approximately 500 mm^3^. Drugs used for treatment included osimertinib (5 mg/kg via oral administration 5 times per week), lorlatinib (6 mg/kg via oral administration 5 times per week), sotorasib (100 mg/kg via oral administration 5 times per week), or anti-CD47 antibody clone B6H12 (250 μg via intraperitoneal injection 3 times per week). The mice were euthanized when the tumor size exceeded 20 mm in any one dimension or when mice reached humane experimental endpoints according to the Institutional Animal Care and Use Committee–approved protocols.

For a syngeneic, immunocompetent tumor model, C57BL/6 mice (The Jackson Laboratory) were engrafted with 3LL ΔNRAS cells or a CD47-KO variant. Mice were engrafted in the subcutaneous tissue using a dual-flank model. Wild-type tumors were engrafted on one flank, and CD47-KO tumors were engrafted on the contralateral flank. Mice were then randomized to treatment with vehicle control or sotorasib (30 mg/kg via oral administration 5 times per week). Tumor volumes were measured twice weekly using electronic calipers and calculated using the formula as above.

### Statistics.

Data analysis of FDA drug screens was performed across 5 replicates of the experiments as follows. GFP^+^ area was quantified from all wells as described above. After logarithmic transformation, row, column and plate effects were controlled using Tukey’s median polish, after which *P* values for individual compounds (Figure 1C) were computed using a 2-tailed 1-sample Student’s *t* test (46). For plate-based time-series measurements (Figure 1D), “relative area” was defined as the GFP^+^ area at time *t* divided by the GFP^+^ area at time 0, obviating the need for controlling row, column, and plate effects. To assess the effect of combining macrophage+anti-CD47 conditions with different classes of drugs (Figure 1E), each class was compared with control wells (i.e., empty or DMSO) using a 2-tailed 2-sample Student’s *t* test with the Benjamini-Hochberg method (47) to control for multiple hypothesis testing.

In general, flow cytometry data were analyzed by comparing geometric mean fluorescence intensity of individual surface antigens by 1-way ANOVA with Holm-Šidák multiple comparison test. Comprehensive surface immunophenotyping was analyzed from a single set of measurements. Data for naive parental versus sotorasib-resistant lines were uniformly adjusted by linear transformation to correct for negative values. Data were fit using linear regression, which was used to define the 95% prediction interval.

In vitro phagocytosis assays were analyzed by comparing the mean percentage of GFP^+^ macrophages among different conditions. Experiments were performed in at least 2 independent trials, using a total of 6–8 individual donors, except as otherwise indicated. The mean values for relevant comparisons were assessed by 2-way ANOVA with Holm-Šidák multiple comparison test using Prism version 9.2.0 (Graphpad).

Long-term coculture assays were performed by measuring GFP^+^ area. Data were analyzed by comparing mean GFP^+^ area between relevant conditions using 1-way ANOVA with Holm-Šidák multiple comparison test. Experiments were performed in 2 independent trials, using a minimum of 6–8 donors, except as otherwise indicated. IC_50_ values were calculated by fitting data to sigmoidal dose-response curve using Prism version 9.2.0 (Graphpad).

Analysis of mouse xenograft tumor models was performed by 2-tailed unpaired *t* test of targeted therapy cohorts versus combination therapy cohorts on the last day of tumor measurements using Prism version 9.2.0 (Graphpad). For dual-flank model of 3LL ΔNRAS, statistical analysis was performed by comparing median tumor volume by 2-tailed paired *t* test using Prism version 9.2.0 (Graphpad).

For all experiments, *P* < 0.05 was considered statistically significant.

### Study approval.

The use of primary lung cancer specimens, patient-derived cell lines, and patient-derived xenografts was performed with specimens collected from patients with written informed consent prior to participation under protocols approved by the Dana-Farber/Harvard Cancer Center Institutional Review Board. Experiments with primary human macrophages were exempt from review by the MIT Committee on the Use of Humans as Experimental Subjects because the cells derived from discarded blood products from anonymous donors. All animal studies were conducted in accordance with the guidelines as published in the *Guide for the Care and Use of Laboratory Animals* (National Academies Press, 2011) and were approved by the Institutional Animal Care and Use Committee of Massachusetts General Hospital or the Massachusetts Institute of Technology Committee on Animal Care.

### Data availability.

Data underlying the figures presented are provided and preserved by means of the Supporting Data Values file that accompanies this manuscript or can be made available from the corresponding author upon request.

## Author contributions

KW and ANH conceptualized the study. KV, JA, TWW, A Maoz, SR, JV, DY, A Meglan, JR, JB, NP, GWB, ANH, and KW contributed methodology and performed analysis. KV, JA, TWW, A Maoz, SR, JV, DY, A Meglan, JR, JB, NP, and KW performed experimentation. KV, JA, A Maoz, SR, JV, TWW, GWB, ANH, and KW provided visualization. KW and ANH acquired funding. KV, JA, GWB, KW, and ANH provided project administration. GWB, KW, and ANH provided supervision. KW wrote the original draft of the manuscript. KV, JA, A Maoz, SR, JV, TWW, DY, GWB, ANH, and KW reviewed and edited the manuscript. The order of co–first authors was designated based on the duration of time each author contributed to this study.

## Supplementary Material

Supplemental data

Supplemental video 1

Supplemental video 2

Supplemental video 3

Supplemental video 4

Supporting data values

## Version 1 03/14/2024

In-Press Preview

## Version 2 05/01/2024

Electronic publication
