# Supplementary material for: Targeted therapies prime oncogene-driven lung cancers for macrophage-mediated destruction
Source: J Clin Invest. 2024 Mar 14;134(9):e169315. doi: 10.1172/JCI169315 (PMC11060739; doi:10.1172/JCI169315)
Supplement: Supplemental data [file jci-134-169315-s113.pdf]

SUPPLEMENTAL MATERIALS

Targeted therapies prime oncogene-driven lung cancers for macrophage-mediated destruction

Kyle Vaccaro[1†], Juliet Allen[1†], Troy W. Whitfield[1], Asaf Maoz[1,2], Sarah Reeves[3], José Velarde[1], Dian Yang[1], Anna Meglan[1], Juliano Ribeiro[1], Jasmine Blandin[1], Nicole Phan[3], George W. Bell[1], Aaron N. Hata[3], and Kipp Weiskopf[1,2,4]*

List of Supplemental Materials

Supplemental Methods

Supplemental Figures 1-20

Supplemental Tables 1-2

Supplemental Movies 1-4

Supplemental Methods

TCGA analysis: Expression levels (RNA-seq HTSeq - FPKM-UQ for selected genes) and mutation status (*EGFR* and *KRAS*) were downloaded from the TCGA LUAD dataset using the UCSC Xena browser. For each mutated gene, samples with an identifiable activating mutation or no identifiable activating mutation ("None") were partitioned and compared with the Welch Two Sample t-test.

Synergy analysis: The MuSyC model (35) for synergy in combination drug therapy was used to detect and characterize synergies in pairwise combinations of targeted therapies and macrophage-activating antibodies. The formalism for the MuSyC model is based upon mass action equations for a two-drug system and when fitted yields parameters that delineate synergies in efficacy (β), potency (α_{12},α_{21}) and cooperativity (γ_{12},γ_{21}). Data that was used to fit the model was generated from long-term co-culture assays using primary human macrophages and GFP+ cancer cells performed in 384-well plates. The conditions in each well reflected dual titrations of targeted therapies and antibodies. The area of GFP signal after 6.5 days, relative to its initial level, was used to measure an "effect" for each well (i.e. the fraction of cells that survive after 6.5 days) and was scaled to be in [0,1]. These per-well "effects" were fitted to the MuSyC model using the synergy Python library.

Cytokine analysis: Primary human macrophages and human cancer cells (PC9 or NCI-H358) were cultured by themselves or together. For each culture condition, the cells were subjected to treatment with vehicle control (PBS), targeted therapy (osimertinib for PC9, sotorasib for NCI-H358), an anti-CD47 antibody, or the combination of the appropriate targeted therapy with an anti-CD47 antibody (combo treatment). Three replicates were performed for each treatment condition, and cells were cultured for a total of 6 days. At the end of the culture period, the

supernatants were collected and frozen at -80°C. The supernatants were subjected to addressable laser bead immunoassay analysis (ALBIA) using a Human Cytokine 71-Plex Discovery Assay (Eve Technologies) to determine cytokine concentrations. For data analysis, values below the calibration range were set to 0, whereas those above the calibration range were set to the maximum calculated value for that cytokine. Statistical comparison of groups was performed with ANOVA and the Dunnett test, using the combo treatment as the reference group. Samples were partitioned into 6 sets of comparisons of interest based on each cell condition as follows: Comparison A (PC9 alone + PBS|osimertinib|anti-CD47|combo); Comparison B (Macrophages alone + PBS|osimertinib|anti-CD47|combo); Comparison C (Macrophages + PC9 co-culture + PBS|osimertinib|anti-CD47|combo); Comparison D (NCI-H358 alone + PBS|sotorasib|anti-CD47|combo); Comparison E (Macrophages alone + PBS|sotorasib|anti-CD47|combo); Comparison F (Macrophages + NCI-H358 co-culture + PBS|sotorasib|anti-CD47|combo). For each comparison, cytokines of interest were chosen where all Dunnett test adjusted p-values were less than 0.05 and the level of the combo treatment group was either higher or lower than all of the other groups. Clustering was performed with Cluster 3.0 and visualized with the pheatmap R package.

Myeloid transcriptional profiling: Primary human macrophages were cultured alone or together with GFP+ NCI-H358 cells. For each cell condition, the samples were then exposed to control treatment or the combination of 10 ug/mL anti-CD47 and 1 uM sotorasib for 4 days. After 4 days, cells from the co-culture were stained with an APC anti-CD45 antibody (BioLegend) and sorted into purified populations of macrophages (CD45+) or NCI-H358 (CD45-) cells. Replicates were kept separate as independent samples. Samples containing macrophages alone were not subjected to sorting. The cell specimens were then lysed with QIAzol and RNA was extracted using miRNeasy Kits (Qiagen) according to the manufacturer's instructions. RNA samples were processed using an nCounter SPRINT Profiler from Nanostring Technologies, according to

manufacturer's directions. Briefly, 50 ng of total RNA derived from co-cultured cells was hybridized with the nCounter Myeloid Innate Immunity Panel at 65°C for 18 hours, before being processed on a SPRINT cartridge. Gene counts from Nanostring expression profiles were normalized with spike-ins (from ERCC library), and statistical comparison of groups (n=2 per group) was performed with ANOVA and the Dunnett test, using the combo treatment as the reference group. Genes of interest were chosen where all Dunnett test adjusted p-values were less than 0.05 and the level of the combo group was either higher or lower than all of the other groups. Clustering was performed with Cluster 3.0 and visualized with the pheatmap R package.

A

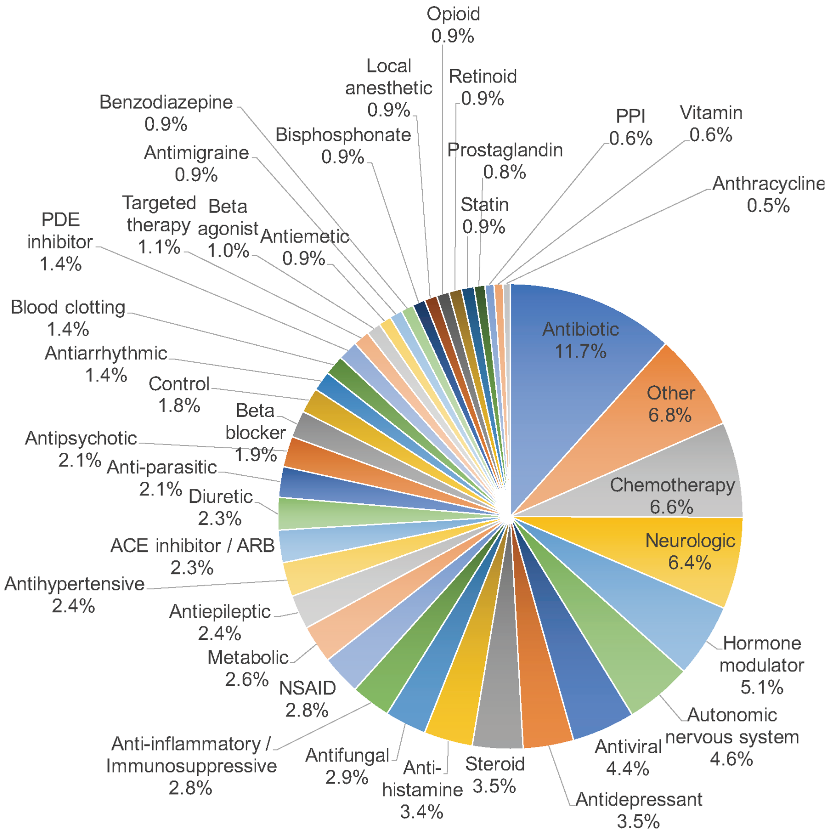

B

**Drug Class**	**N**	**%**
Antibiotic	93	11.6%
Other	54	6.8%
Chemotherapy	53	6.6%
Neurologic	51	6.4%
Hormone modulator	41	5.1%
Autonomic nervous system	37	4.6%
Antiviral	35	4.4%
Antidepressant	28	3.5%
Steroid	28	3.5%
Anti-histamine	27	3.4%
Antifungal	23	2.9%
Anti-inflammatory / Immunosuppressive	22	2.8%
NSAID	22	2.8%
Metabolic	21	2.6%
Antiepileptic	19	2.4%
Antihypertensive	19	2.4%
ACE inhibitor / ARB	18	2.3%
Diuretic	18	2.3%
Anti-parasitic	17	2.1%
Antipsychotic	17	2.1%
Beta blocker	15	1.9%
Control	14	1.8%
Antiarrhythmic	11	1.4%
Blood clotting	11	1.4%
PDE inhibitor	11	1.4%
Targeted therapy	9	1.1%
Beta agonist	8	1.0%
Antiemetic	7	0.9%
Antimigraine	7	0.9%
Benzodiazepine	7	0.9%
Bisphosphonate	7	0.9%
Local anesthetic	7	0.9%
Opioid	7	0.9%
Retinoid	7	0.9%
Statin	7	0.9%
Prostaglandin	6	0.8%
PPI	5	0.6%
Vitamin	5	0.6%
Anthracycline	4	0.5%
EGFR TKI	2	0.3%
**Total**	**800**	**100%**

Supplemental Figure 1

Supplemental Figure 1: The composition of an FDA-approved drug library used for screening efforts. (**A**) Chart depicting drug classes included in the screening library. Percentages indicate number of drugs per class from a total of *N* = 800 individual drug wells and including 14 DMSO controls. (**B**) Table depicting the number and percentage of drugs from each class included in the screening library.

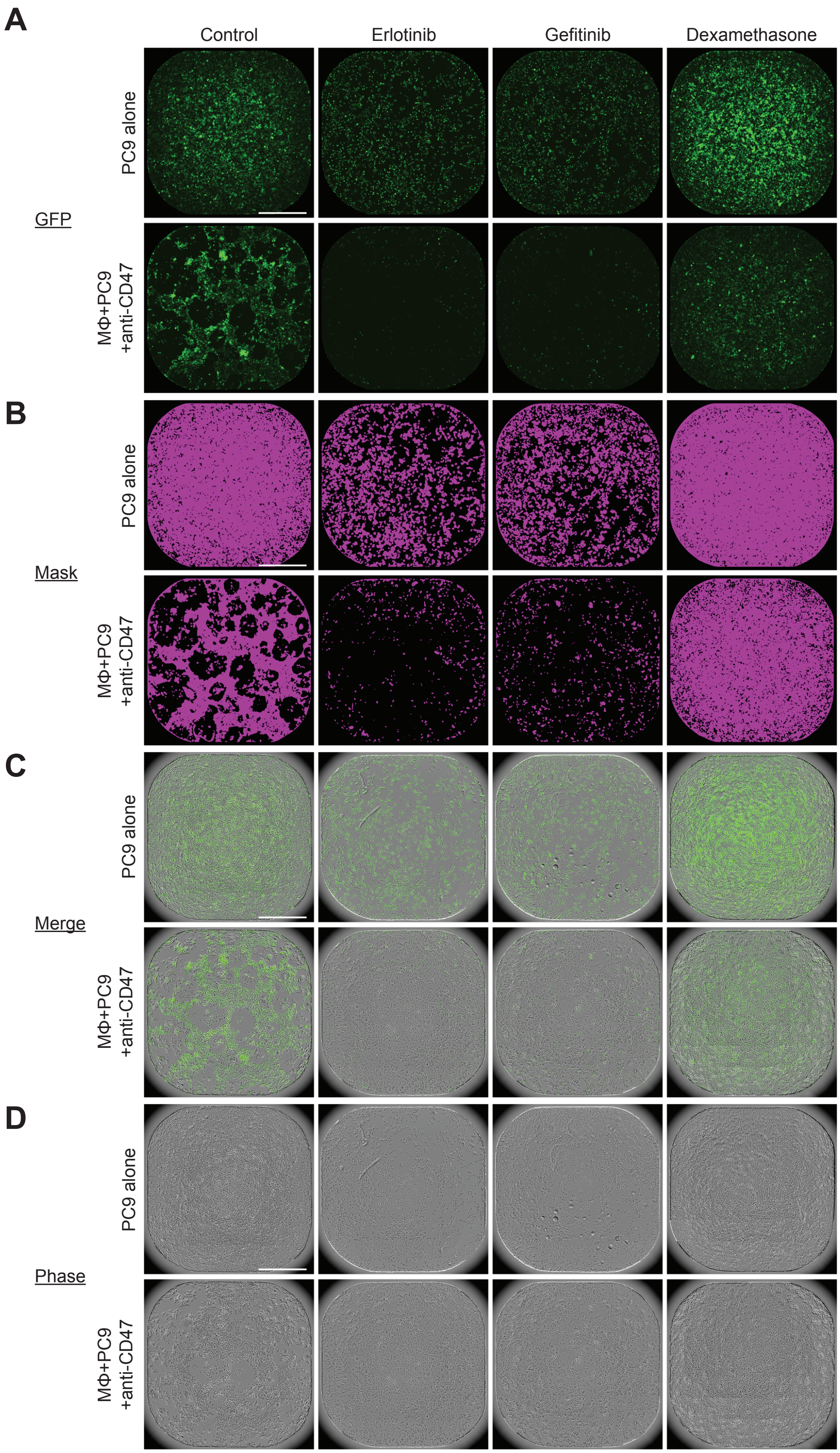

Supplemental Figure 2

Supplemental Figure 2: Representative images of wells from small molecule screen using FDA-approved drug library. GFP+ PC9 cells were combined with primary human macrophages and the indicated drug therapies in 384-well plates. Representative images are shown from a single experimental run of the full FDA-approved drug library using macrophages derived from an individual blood donor at t = 3d 16h. (**A**) Whole well imaging of the GFP+ channel from wells treated with the indicated therapies. Erlotinib and gefitinib were identified as drugs that enhance macrophage-dependent cytotoxicity of PC9 cells, while dexamethasone and other steroid compounds were identified as inhibitors of macrophage-dependent cytotoxicity. (**B**) Image mask of GFP+ pixels (purple) used for quantification and analysis. Note that this panel is repeated from Figure 1B for visual comparison to other images in this figure. (**C**) Overlay of GFP+ channel with phase contrast imaging. (**D**) Phase contrast imaging showing confluency of wells with GFP+ PC9 cells and primary human macrophages present.

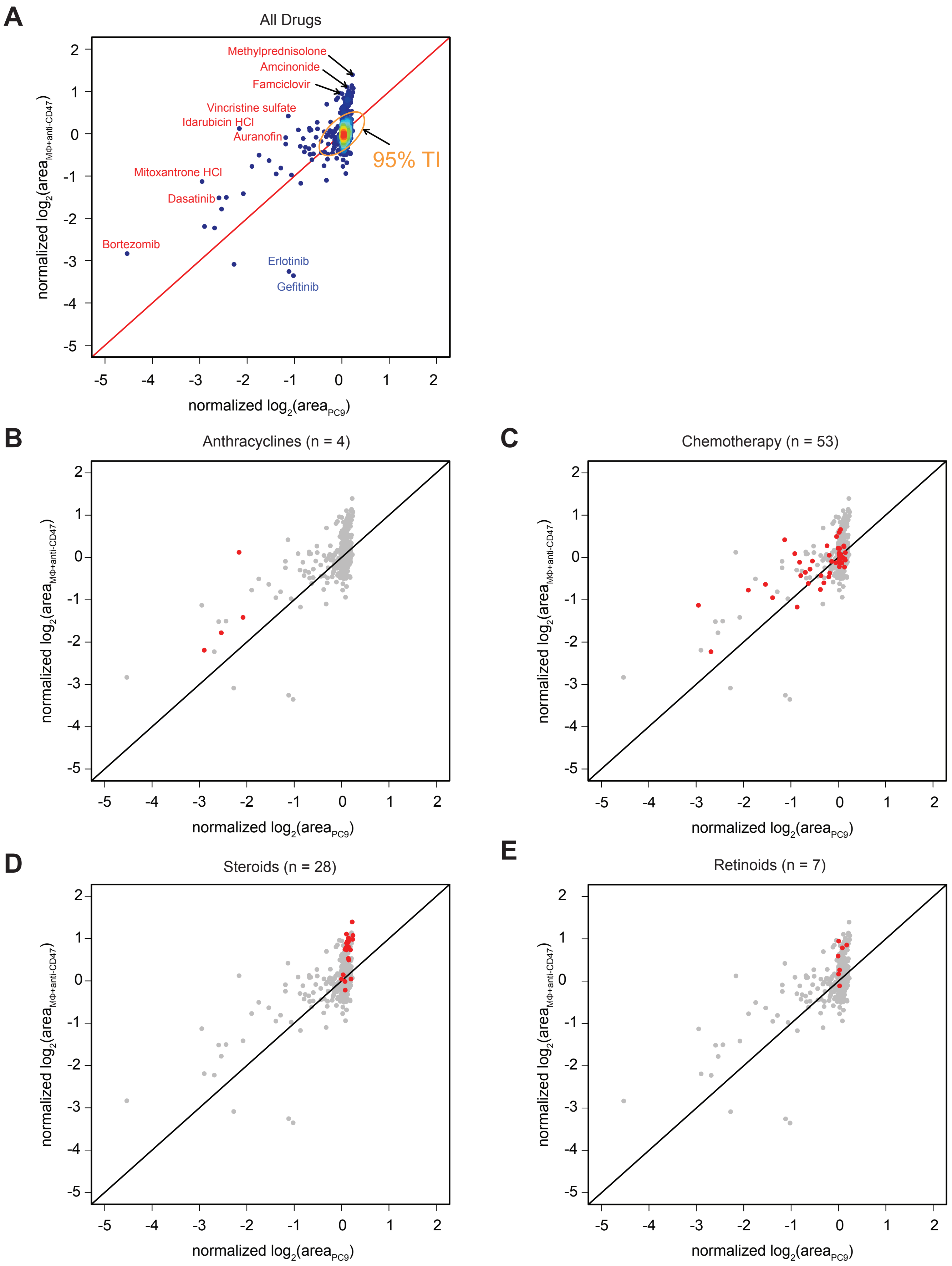

Supplemental Figure 3

Supplemental Figure 3: Analysis of high-throughput screen reveals differential activity of drugs from the FDA-approved library. (**A**) Scatter plot showing how drugs affect growth of GFP+ PC9 cells alone (x-axis) versus when they are co-cultured with macrophages and anti-CD47 therapy (y-axis). Points are colored by density from low (blue) to high (red), with the majority of drugs localized near the origin, indicating no activity affecting either condition. The red diagonal identity line indicates where drugs affect PC9 cells equally under both treatment conditions. The majority of drugs have no significant effect under either condition. The color-coded density gradient represents 95% of drugs with outliers shown as blue dots. The indicated 95% tolerance interval (TI) was constructed after fitting the joint density to a single two-dimensional Gaussian distribution. Drugs indicated in blue or red were identified as hits based on statistical significance and >2-fold change in effect size between the two treatment conditions (see **Fig. 1C**). Drugs highlighted in blue (erlotinib, gefitinib) significantly enhanced macrophage-dependent killing of GFP+ PC9 cells. Drugs highlighted in red resulted in more cancer cell growth of PC9 cells in the presence of macrophages+anti-CD47 therapy versus the PC9 alone condition. However, drugs within this category exhibited different effects. Anthracyclines (**B**) and other chemotherapy drugs (**C**) exerted direct cytotoxicity to the PC9 cells alone, but the addition of macrophages+anti-CD47 protected the cancer cells from these drugs. In contrast, steroids (**D**) and retinoids (**E**) inhibited macrophage-mediated killing of the PC9 cells, thereby resulting in relatively enhanced growth in the macrophage+anti-CD47 condition. (B-E) Highlighted classes of drugs are show in red.

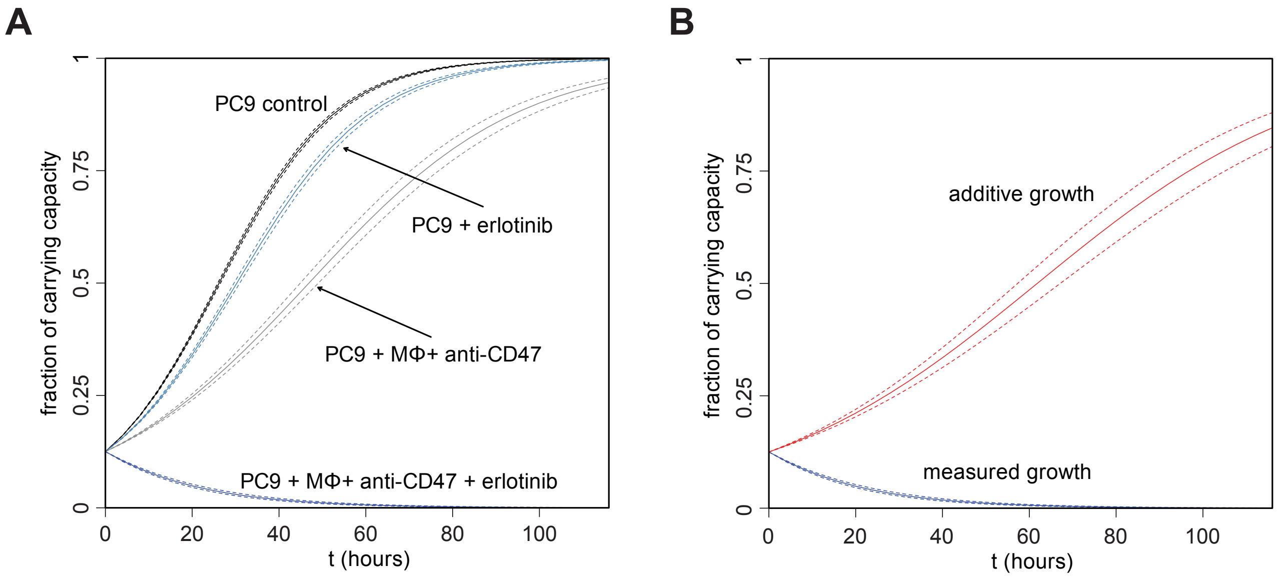

Supplemental Figure 4

Supplemental Figure 4: Modeling additive growth reveals synergy between EGFR inhibitors and anti-CD47 therapy. (**A**) Growth rates were estimated by fitting logistic growth models to time series data collected on PC9 cells grown under various conditions. Because the presence of macrophages with anti-CD47 antibodies elicits an anti-tumor response that limits the carrying capacity of PC9 cultures, the plots for fitted growth are rendered in terms of cell populations (i.e. area of GFP+ cells) normalized to carrying capacity. (**B**) The estimated growth rates were used to construct a purely additive model (i.e. without any synergy or antagonism) for the combined effects of erlotinib and macrophages + anti-CD47 antibodies. The dashed lines for each curve indicate the standard errors in the fitting. The dramatic reduction observed in the growth rate of PC9 cells due to the combination of erlotinib with macrophages + anti-CD47 antibodies is far greater than that predicted under the assumption of additivity, indicating that cooperativity is present.

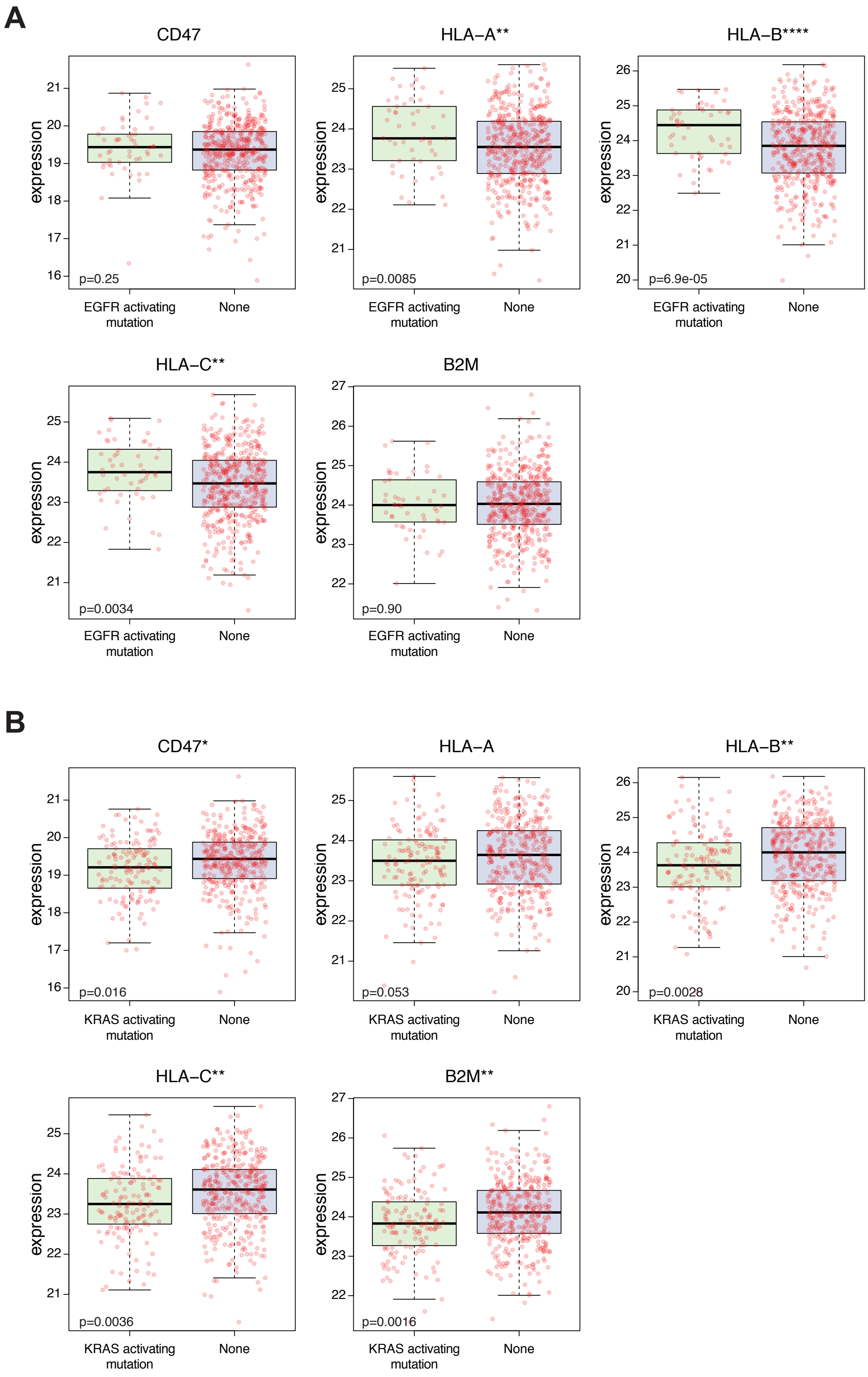

Supplemental Figure 5

Supplemental Figure 5: Expression analysis of lung adenocarcinoma specimens from the TCGA database. Expression data for lung adenocarcinoma (LUAD) specimens was downloaded from the TCGA database (n=509). Samples were categorized as containing activating mutations in *EGFR* or *KRAS* based on published literature. Expression of *CD47*, *HLA-A*, *HLA-B*, *HLA-C*, and *B2M* were examined. (**A**) RNA-seq expression (HTSeq - FPKM-UQ) of selected genes in TCGA LUAD samples, comparing those with identifiable activating mutations in *EGFR* (n=50) to those with no identifiable activating mutation (n=459) using Welch's (two-sample) t-test. *HLA-A*, *HLA-B*, *HLA-C* exhibited significantly higher levels of expression in specimens with *EGFR* activating mutations versus those with no identifiable activating mutation. (**B**) RNA-seq expression (HTSeq - FPKM-UQ) of selected genes in TCGA LUAD samples, comparing those with activating mutations in *KRAS* (n=139) to those with no mutation (n=370) using Welch's (two-sample) t-test. *CD47*, *HLA-B*, *HLA-C*, and *B2M* were significantly lower in expression in specimens containing a *KRAS* activating mutation compared to those specimens with no identifiable *KRAS* activating mutation (None). (**A-B**) *p<0.05, **p<0.01, ****p<0.0001 for the indicated comparisons.

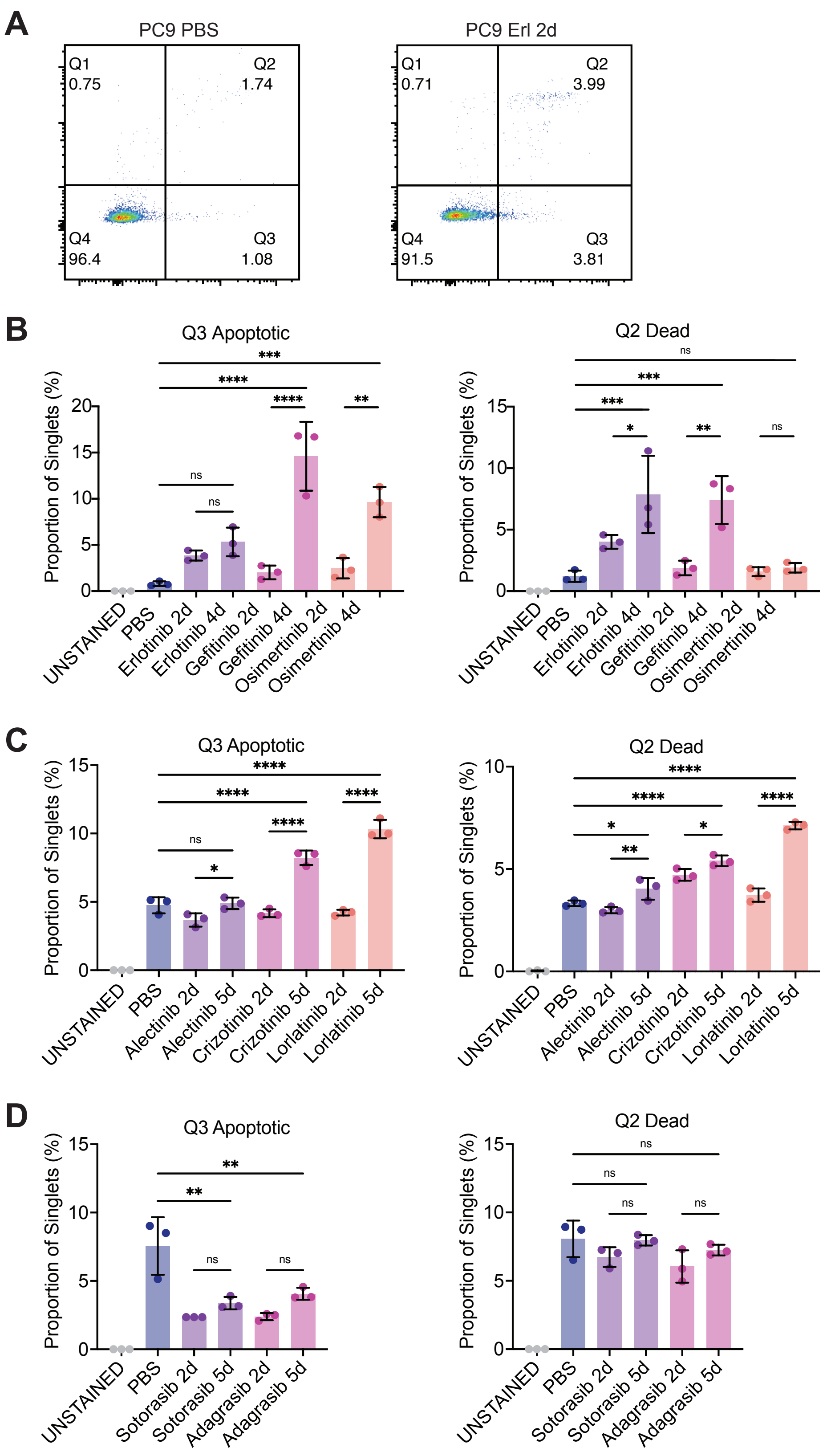

Supplemental Figure 6

Supplemental Figure 6: Analysis of apoptosis and cell death in response to targeted therapies. Lung cancer cells were treated with the indicated targeted therapies for 2-5 days. Adherent cells were collected and analyzed by flow cytometry for viability and apoptosis using annexin V and DAPI. (**A**) Representative plots showing PC9 cells treated with vehicle control or erlotinib to demonstrate gating strategy. Gates were drawn to quantify apoptotic cells (annexin V+, DAPI-) and dead cells (annexin V+, DAPI+). (**B**) Quantification of the percentage of PC9 cells undergoing apoptosis or cell death in response to the indicated EGFR TKIs. (**C**) Quantification of the percentage of NCI-H3122 cells undergoing apoptosis or cell death in response to the indicated ALK TKIs. (**D**) Quantification of the percentage of NCI-H358 cells undergoing apoptosis or cell death in response to the indicated $KRAS^{G12C}$ inhibitors. (B-D) Data represent mean ± SD from 3 replicates performed from one experiment. ns, not significant, *p<0.05, **p<0.01, ***p<0.001, ****p<0.0001 by one-way ANOVA with Tukey's multiple comparisons test.

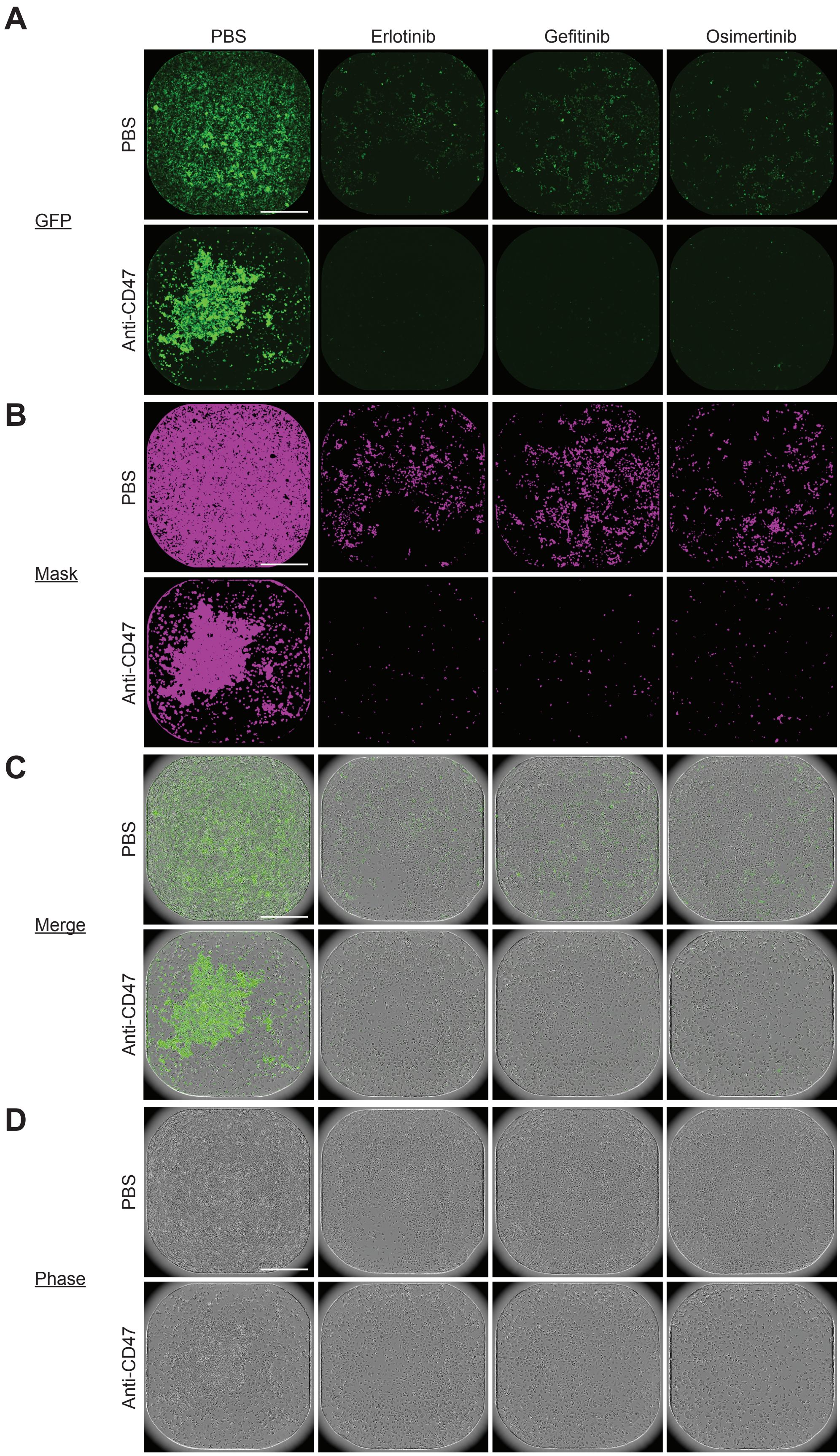

Supplemental Figure 7

Supplemental Figure 7: Representative images of long-term co-culture assays using GFP+ PC9 cells and human macrophages. GFP+ PC9 cells were combined with primary human macrophages and the indicated drug therapies in 384-well plates. Representative images are shown from a macrophages derived from an individual blood donor at t = 6d 12h. (**A**) Whole well imaging of the GFP+ channel from wells treated with the indicated therapies. (**B**) Image mask of GFP+ pixels (purple) used for quantification and analysis. Note that this panel is repeated from Figure 3A for visual comparison to other images in this figure. (**C**) Overlay of GFP+ channel with phase contrast imaging. (**D**) Phase contrast imaging showing confluency of wells with GFP+ PC9 cells and primary human macrophages present.

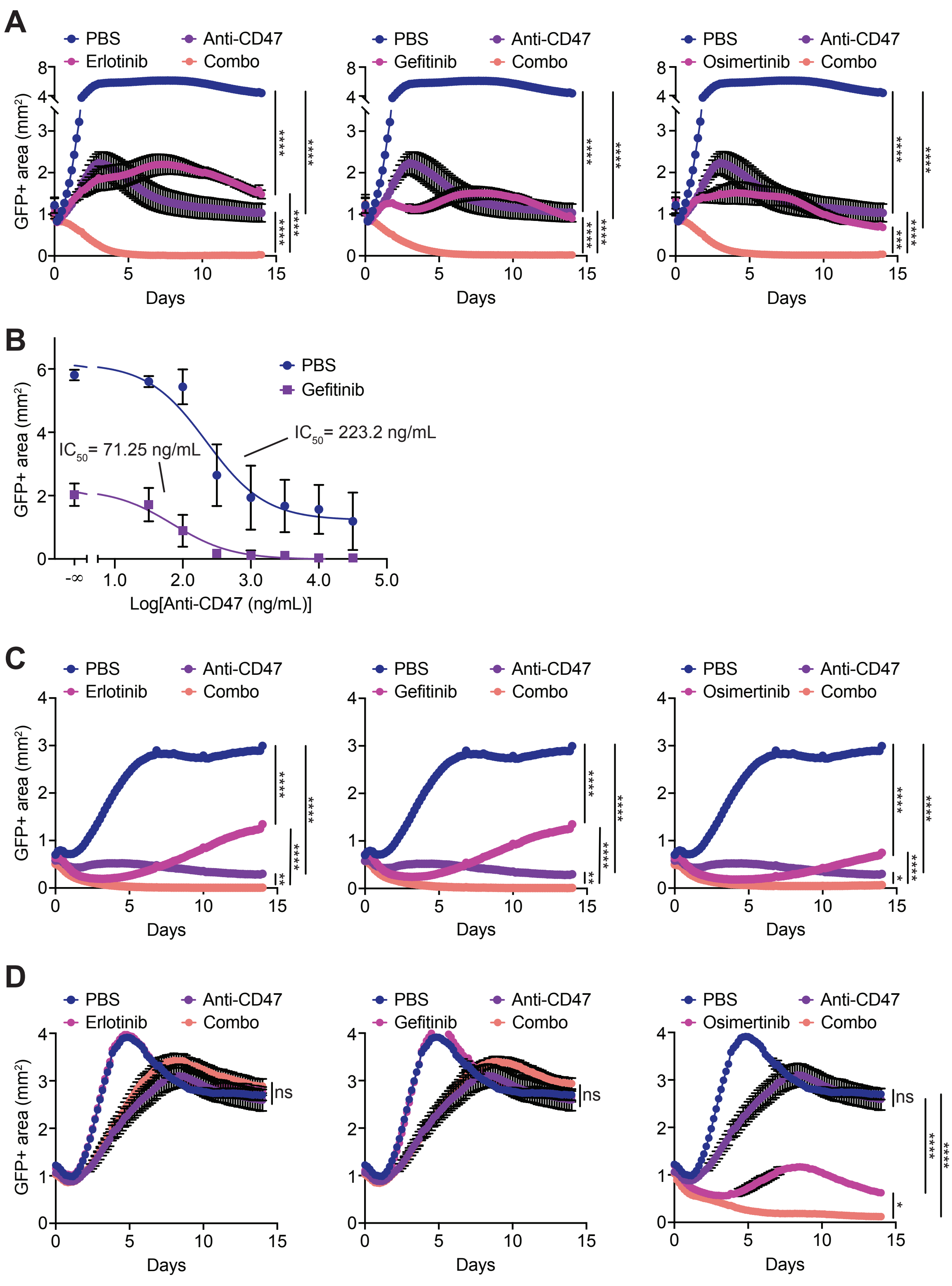

Supplemental Figure 8

Supplemental Figure 8: Growth curves of long-term assays using human macrophages and different *EGFR* mutant lung cancer specimens. GFP+ lung cancer cells were combined with primary human macrophages and the indicated drug therapies in 384-well plates. The GFP+ area, representing the growth or death of the GFP+ cancer cells, was evaluated by whole-well imaging every 4 hours and quantified by automated image analysis. (**A**) GFP+ PC9 cells co-cultured with macrophages and erlotinib (left), gefitinib (middle), or osimertinib (right). Note curves are repeated from Figure 3B for reference. (**B**) Co-culture assays using GFP+ PC9 cells and human macrophages to evaluate a dose-response relationship. The concentration of anti-CD47 was titrated alone or in combination with gefitinib at 100 nM. The IC_{50} for anti-CD47 improved from 223.2 ng/mL (95% CI 158.2-317.3) to 71.25 ng/mL (95% CI 52.39-97.22). GFP+ area measured and compared on day 6.5 of co-culture. (**C**) GFP+ MGH119 cells co-cultured with macrophages and erlotinib (left), gefitinib (middle), or osimertinib (right). (**D**) GFP+ MGH134 cells co-cultured with macrophages and erlotinib (left), gefitinib (middle), or osimertinib (right). (A-D) Data at each timepoint represent mean ± SEM from 3-4 co-cultures per donor and n = 4-8 independent macrophage donors. ns, not significant, *p<0.05, **p<0.01, ***p<0. 001, ****p<0.0001 by two-way ANOVA with Holm-Sidak multiple comparisons test on day 14 of co-culture or as indicated. Note PBS and Anti-CD47 curves are repeated for reference, and that some targeted therapy and combo curves appear similar but represent different underlying data. These data represent full growth curves from experiments presented in Figure 3A-E.

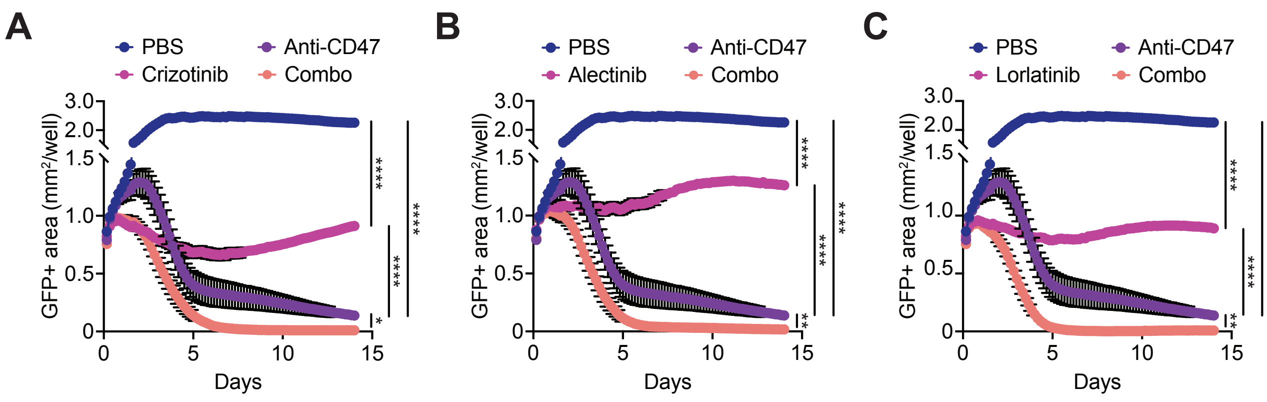

Supplemental Figure 9

Supplemental Figure 9: Growth curves of long-term assays using human macrophages and an *ALK* rearranged lung cancer cell line. GFP+ NCI-H3122 lung cancer cells were combined with primary human macrophages and the indicated drug therapies in 384-well plates. The GFP+ area, was evaluated by whole-well imaging every 4 hours and quantified by automated image analysis. GFP+ NCI-H3122 cells co-cultured with macrophages and crizotinib (**A**), alectinib (**B**), or lorlatinib (**C**). Data at each timepoint represent mean ± SEM from 3 technical co-cultures per donor using n = 4 independent macrophage donors. ****p<0.0001 by two-way ANOVA with Holm-Sidak multiple comparisons test on day 14 of co-culture. Note PBS and Anti-CD47 curves are repeated for reference. These data represent full growth curves from experiments presented in Figure 4A.

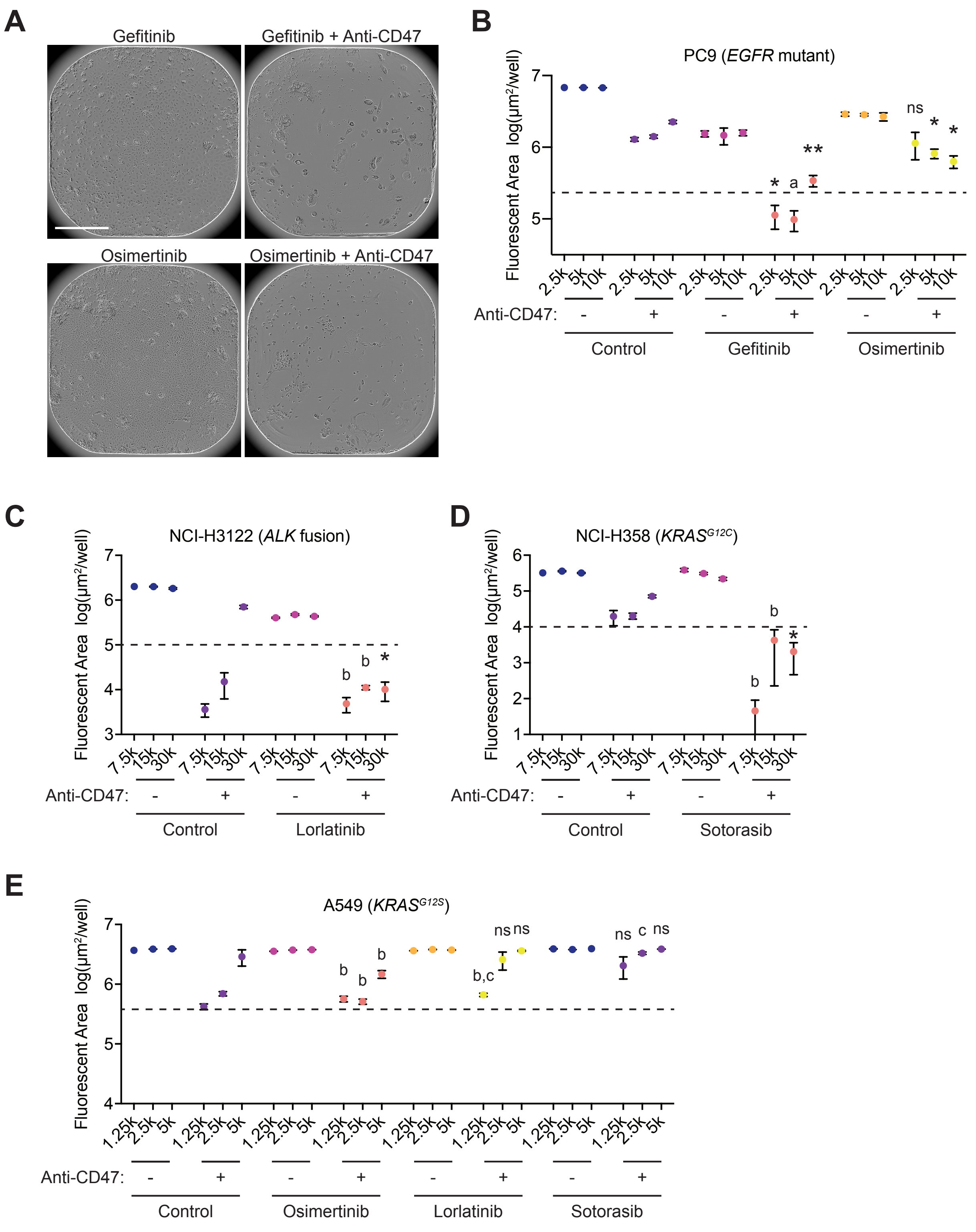

Supplemental Figure 10

Supplemental Figure 10: The combination therapy reduces persister cell numbers to prevent their regrowth. Cancer cells were co-cultured with macrophages and the indicated drugs for 14 days. For each cell line, three different cancer cell numbers were tested, representing 1x, 2x, or 4x the standard number of cells used per well (in thousands, k) with a constant number of macrophages (10,000 cells). On day 14, the drugs were washed out and fresh medium was added. Co-cultures were continued for 9 additional days to allow persister cells to regrow. (**A**) Representative phase images after drug washout showing confluency and regrowth of PC9 cells treated with single-agent EGFR TKIs compared to the combination therapy, which primarily shows residual, enlarged macrophages. Scale bar, 800 μm. (**B-D**) Quantification of persister cell regrowth on day 9 after washout using GFP+ PC9 cells (**B**), GFP+ NCI-H3122 cells (**C**), or GFP+ NCI-H358 cells (**D**) in response to the indicated therapies. (**E**) As a negative control, mCherry+ A549 lung cancer cells were also tested. These cells contain a $KRAS^{G12S}$ mutation but lack an actionable driver mutation. (**B-E**) Dashed line represents approximate threshold of background versus persister cell regrowth based on visual inspection of wells. Statistical analysis was performed by Brown-Forsythe and Welch ANOVA for the combination therapy relative to each single-agent therapy for each respective cancer cell number. Corrections were performed for multiple comparisons. ns, not significant for the indicated combination therapy condition relative to either single-agent control; $^*p<0.05$, $^{**}p<0.01$ for the indicated combination therapy condition relative to both single-agent controls; $^{a}p<0.05$ for the indicated combination therapy condition relative to the respective anti-CD47 treatment only; $^{b}p<0.05$ for the indicated combination therapy condition relative to the respective targeted therapy only, $^{c}p<0.05$ for the indicated combination therapy condition relative to the respective anti-CD47 treatment only but with opposite effect.

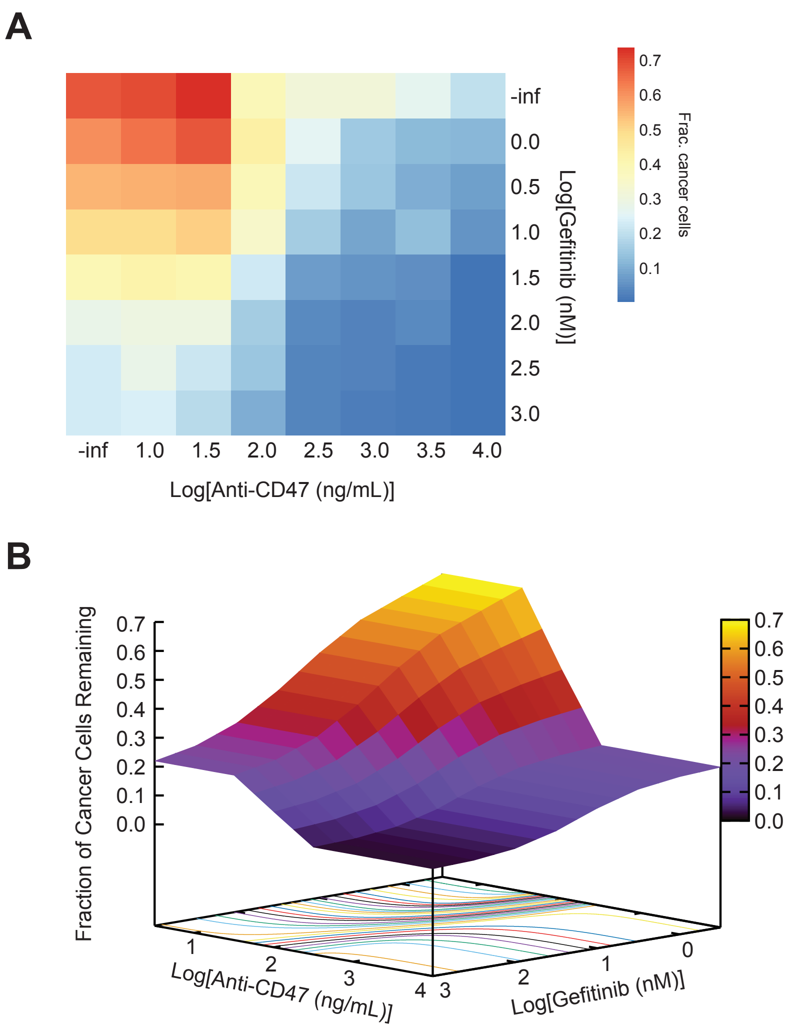

Supplemental Figure 11

Supplemental Figure 11: Targeted therapies synergize with anti-CD47 antibodies in macrophage co-culture assays. Co-culture assays were performed in which a matrix of drug concentration-combinations was generated for a targeted therapy and an anti-CD47 antibody. For each drug, concentrations were titrated across 8 half-log dilutions. A matrix was generated to generate 64 different concentration combinations. Each of these combinations was tested in long-term co-culture assays with primary human macrophages and the appropriate GFP+ cancer cells. The growth or elimination of the cancer cells was measured by assessing the GFP+ area on day 6.5 of co-culture. The data was subjected to MuSyC analysis to evaluate for synergy with respect to efficacy (β), potency (α_{12}, α_{21}) and cooperativity (γ_{12}, γ_{21}). (**A**) Representative heatmap showing mean effects across replicates for titrations of gefitinib and an anti-CD47 antibody (clone B6H12). (**B**) Representative example of three-dimensional contour plot showing data fitted to the full MuSyC model for gefitinib in combination with an anti-CD47 antibody. Data are summarized from 6 co-cultures per combination condition and represent experiments performed with n = 2 independent macrophage donors (donor 241 and 242).

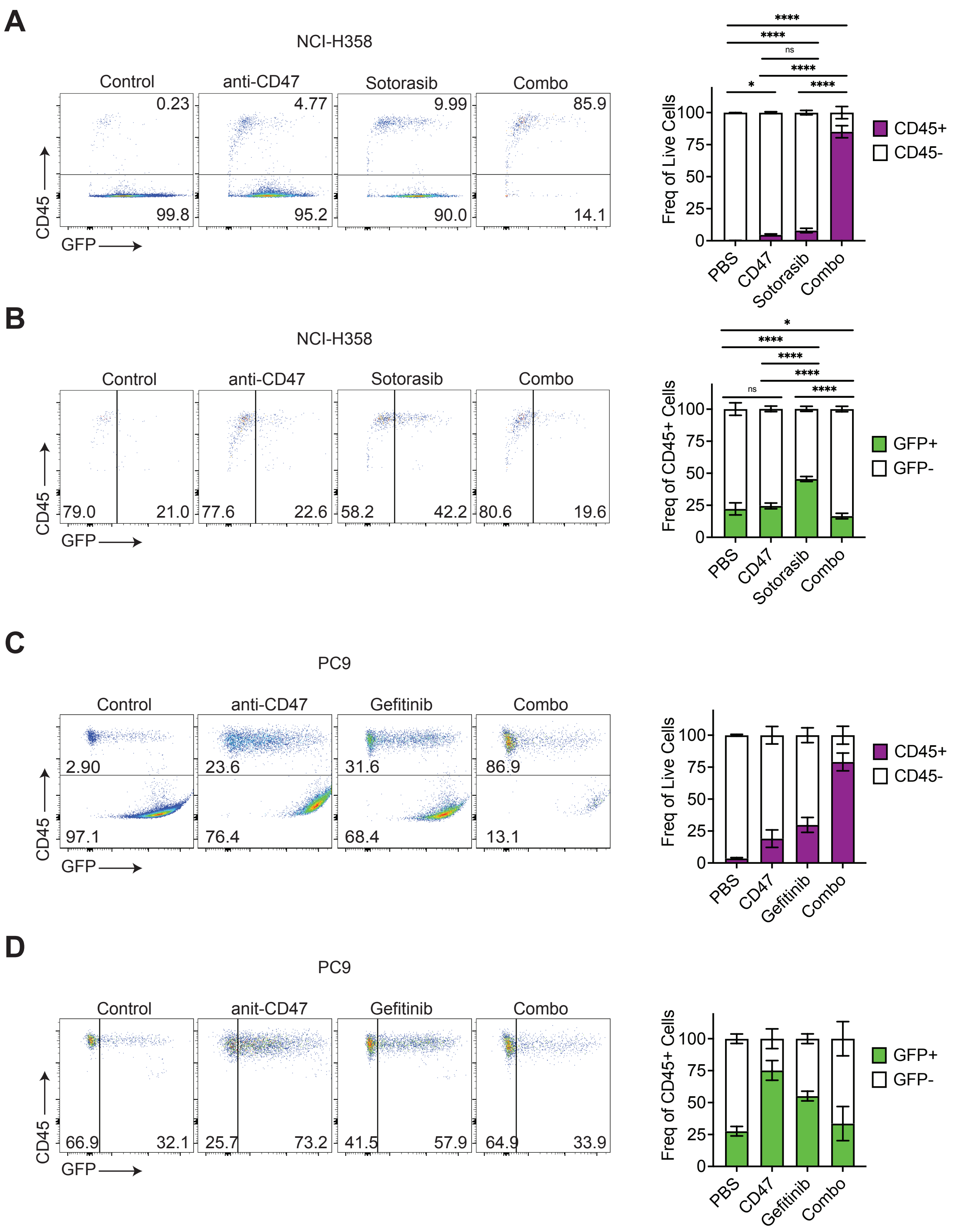

Supplemental Figure 12

Supplemental Figure 12: Flow cytometry analysis of long-term co-cultures assays demonstrates phagocytosis and elimination of cancer cells. Primary human macrophages were co-cultured with GFP+ NCI-H358 cells (**A-B**) or GFP+ PC9 cells (**C-D**) and the indicated therapies. Cells were collected on day 4 of co-culture and analyzed by flow cytometry. Macrophages were identified by APC anti-CD45 and lung cancer cells were identified by GFP fluorescence. The percentage of GFP+ macrophages was quantified as a representation of phagocytosis. An increase in the percentage of CD45+ cells (A, C) was observed due to elimination of cancer cells in the co-culture. Similarly, the percentage of phagocytic macrophages decreased in the combo therapy treatment due to decreases in cancer cell number and digestion of internalized material (B, D). For each condition, representative FACS plots are shown on the left and summary bar graphs depicting mean ± SD are shown on the right. ns, not significant, *$p<0.05$, ****$p<0.0001$ by two-way ANOVA with Tukey's multiple comparisons test from one experiment using macrophages derived from 3 independent donors with 8 co-cultures per donor (PC9). Data presented for NCI-H358 cells performed with 1 donor with 8 co-cultures. Note that the data presented in panels B and D (CD45+ cells) represent a subset of the total live cell populations presented in A and C, respectively.

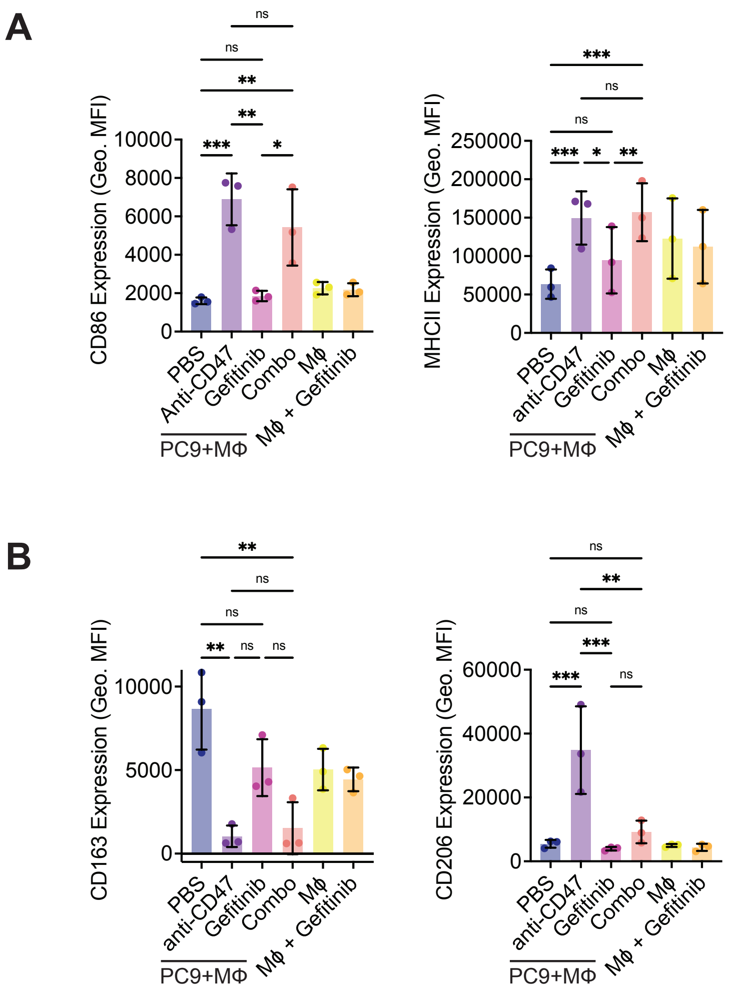

Supplemental Figure 13

Supplemental Figure 13: Analysis of macrophage polarization state following co-culture of macrophages and lung cancer cells. Primary human macrophages were cultured alone or with GFP+ PC9 cells and the indicated therapies. Cells were collected on day 4 of co-culture and analyzed by flow cytometry for the antigens associated with (**A**) M1 polarization (CD86, MHC II); or (**B**) M2 polarization (CD163, CD206). Bar graphs depict mean ± SD from n = 3 independent donors with one technical replicate per donor. ns, not significant, *$p<0.01$, ****$p<0.0001$ by two-way ANOVA with Tukey's multiple comparisons test from one independent experiment.

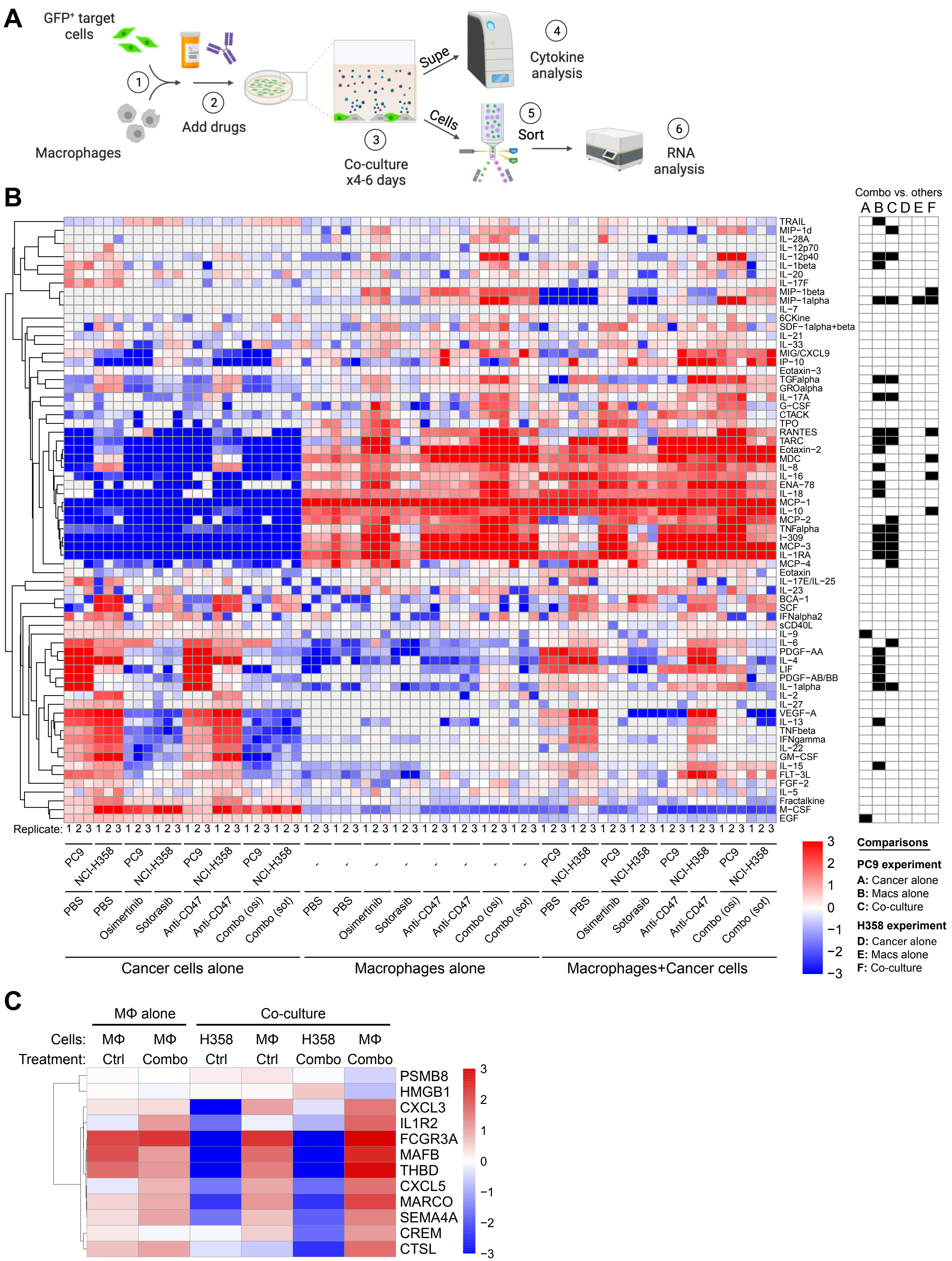

Supplemental Figure 14

Supplemental Figure 14: The combination of targeted therapies and anti-CD47 elicits unique cytokine and gene expression signatures in co-culture assays. (**A**) Diagram showing experimental setup of cytokine and RNA profiling experiments. Primary human macrophages were co-cultured with GFP+ target NSCLC cells (PC9 or NCI-H358) with targeted therapies and/or an anti-CD47 antibody. Cells were co-cultured for 4-6 days. Supernatants were collected and subjected to multiplex cytokine analysis of 71 human analytes by addressable laser bead immunoassay. In a separate experiment, adherent cells were collected, sorted by flow cytometry, and subjected to targeted gene expression profiling of 770 myeloid-derived genes using an nCounter Myeloid Innate Immunity Panel (Nanostring). (**B**) Multiplex cytokine analysis of supernatants from co-culture assays using primary human macrophages and PC9 or NCI-H358 NSCLC cells. Heatmap shows mean-normalized levels of each cytokine in each sample (n=3), clustering log-transformed cytokine levels with uncentered correlation, merging clusters by pairwise average-linkage. Matrix on the right indicates cytokines of interest where all Dunnett test adjusted p-values were less than 0.05 and the level of the combo treatment group was either higher or lower than all of the other groups. The six statistical comparisons (A-F) are further described in the methods. (**C**) Targeted gene expression analysis depicting myeloid-derived genes from co-culture assays of primary human macrophages and NCI-H358 cells. Heatmap indicates mean-normalized levels of each gene in each sample (n=2), clustering genes by log-transformed RNA levels with uncentered correlation, merging clusters by pairwise average-linkage. The indicated genes of interest were identified as those for which all Dunnett test adjusted p-values were less than 0.05 and the level of the combo group was either higher or lower than all of the other groups.

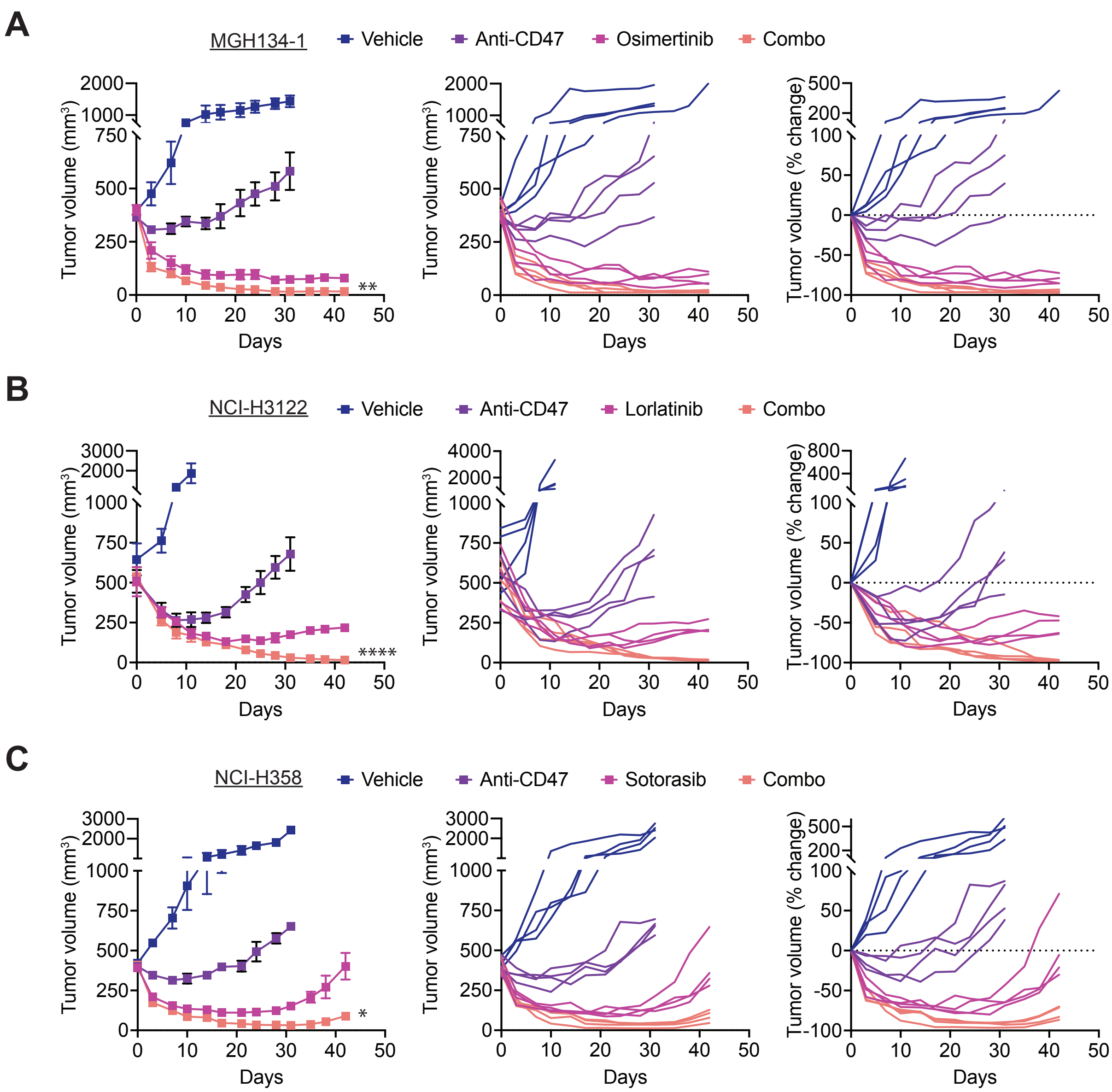

Supplemental Figure 15

Supplemental Figure 15: Growth curves of lung cancer tumors in xenograft treatment experiments. Full growth curves from mouse xenograft tumor models shown in Fig. 5 depicting tumor volumes as mean ± SEM (left), growth curves from individual mice (middle) or fold-change from individual mice. (**A**) GFP+ MGH134-1 cells treated with vehicle control, anti-CD47 alone, osimertinib alone, or the combination (combo). (**B**) GFP+ NCI-H3122 cells treated with vehicle control, anti-CD47 alone, lorlatinib alone, or the combination. (**C**) GFP+ NCI-H358 cells treated with vehicle control, anti-CD47 alone, sotorasib alone, or the combination. (A-C) *$p<0.05$, **$p<0.01$, ***$p<0.001$ for the combination therapy versus targeted therapy by unpaired t test. For each experiment, n = 4 mice per treatment cohort.

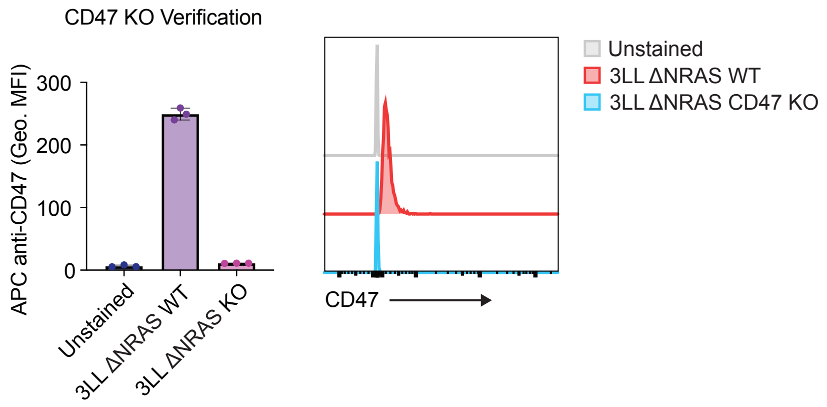

Supplemental Figure 16

Supplemental Figure 16: Validation of a CD47 KO line generated by CRISPR/Cas9 editing of 3LL ΔNRAS cells. Cell-surface CD47 expression as detected by flow cytometry for wild-type (WT) 3LL ΔNRAS cells or a CD47 knockout (KO) variant. Data are depicted as mean ± SD from three replicates (left), or as representative histograms (right).

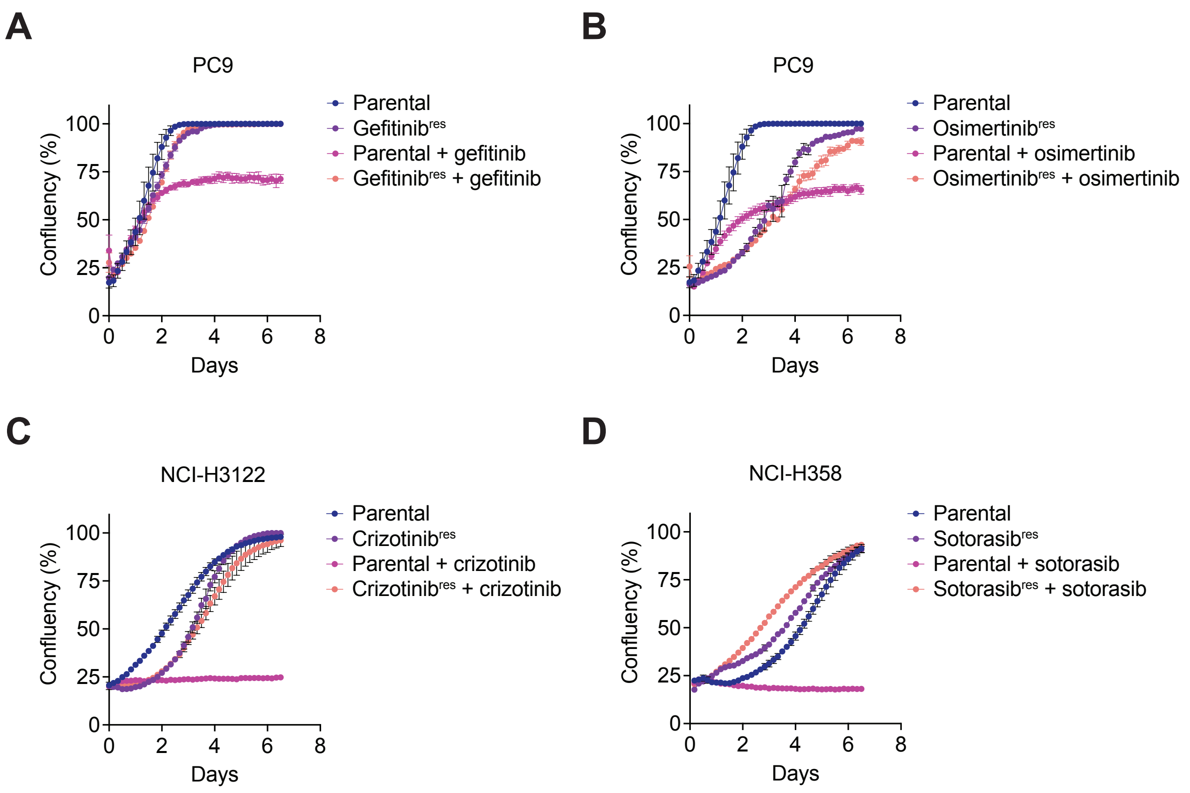

Supplemental Figure 17

Supplemental Figure 17: Proliferation of NSCLC cell lines in vitro after acquiring resistance to targeted therapies. Resistant cell lines were generated by prolonged culture of NSCLC cell lines in appropriate targeted therapy. Proliferation was evaluated by confluency analysis as measured by phase microscopy and automated image analysis. Proliferation was measured without drug selection or with 1 uM targeted therapy as indicated. Cell lines tested included PC9 cells resistant to gefitinib (**A**) or osimertinib (**B**), NCI-H3122 cells resistant to crizotinib (**C**), or NCI-H358 cells resistant to sotorasib (**D**). For the majority of cell lines, growth rates were comparable between parental and resistant cells in the absence of targeted therapy and approached 100% confluency by day 6.5 of culture. Data represent mean of 3 technical replicates ± SEM from one independent experiment for each cell line. (A-B) PC9 evaluation was performed in a single experiment and separated into distinct plots with the same parental curve reproduced for data visualization.

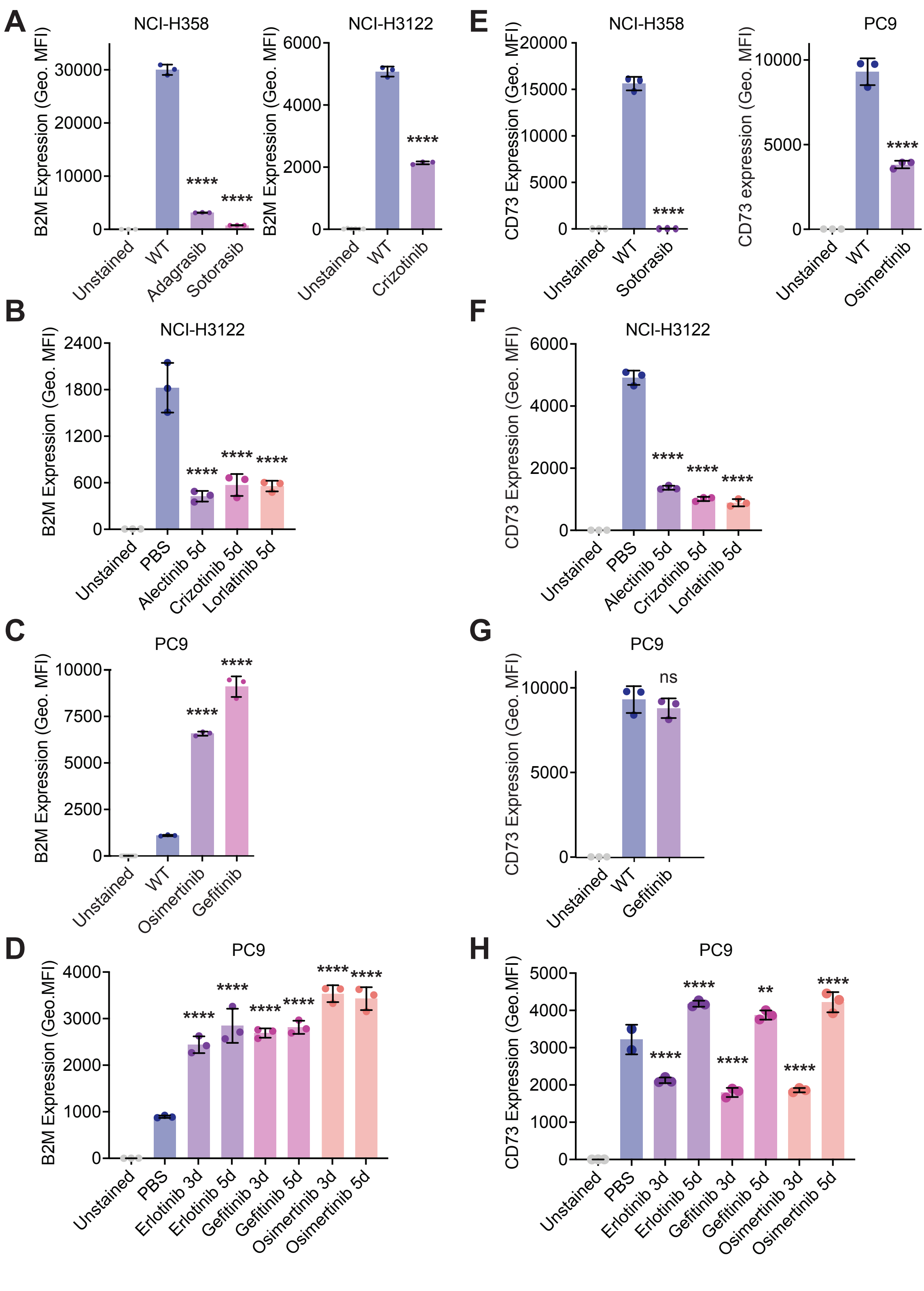

Supplemental Figure 18

Supplemental Figure 18: Changes in B2M and CD73 expression on lung cancer cells exposed to targeted therapies. (**A**) Downregulation of B2M on NCI-H358 cells or NCI-H3122 cells resistant to the indicated targeted therapies. (**B**) Downregulation of B2M on NCI-H3122 cells following treatment with the indicated ALK inhibitors. (**C**) B2M was not downregulated on PC9 cells that are resistant to EGFR inhibitors, nor PC9 cells exposed to EGFR inhibitors in culture (**D**). (**E**) CD73 is downregulated on NCI-H358 and PC9 cells that are resistant to the indicated targeted therapies. (**F**) NCI-H3122 cells downregulate CD73 in response to the indicated targeted therapies. (**G**) PC9 cells resistant to gefitinib did not downregulate CD73. (**H**) CD73 is dynamically regulated on the surface of PC9 cells in response to EGFR inhibitors, with initial downregulation after 3 days of exposure, followed by increased surface expression. (A-H) Data represent mean ± SD from 3 technical replicates from individual experiments. ns, not significant, ****$p<0.0001$ by one-way ANOVA with Holm-Sidak multiple comparisons test.

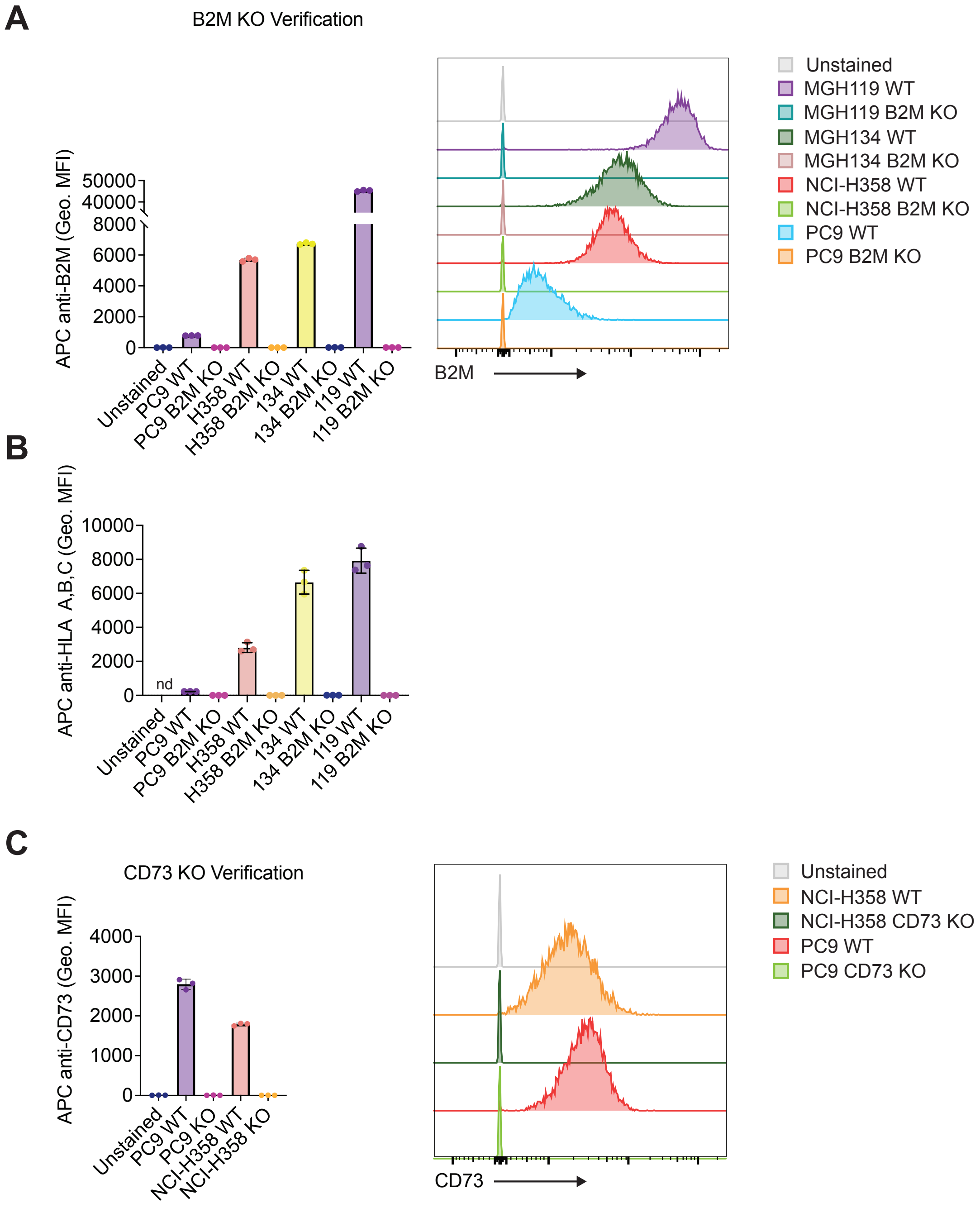

Supplemental Figure 19

Supplemental Figure 19: Validation of B2M KO and CD73 KO lines generated by CRISPR/Cas9 editing of human lung cancer cell lines. (**A**) Flow cytometry analysis of B2M expression on the surface of wild-type (WT) PC9, NCI-H358, MGH134, and MGH119 cells compared to their respective B2M KO variants. Left, quantification of geometric mean fluorescence intensity for B2M surface expression (Geo. MFI). Right, representative histograms showing B2M surface expression. (**B**) Flow cytometry analysis of HLA-A,B,C expression on the surface of wild-type (WT) PC9, NCI-H358, MGH134, and MGH119 cells compared to their respective B2M KO variants. Knockout of B2M abrogates HLA-A,B,C expression. (**C**) Flow cytometry analysis of CD73 expression on the surface of wild-type (WT) PC9 and NCI-H358 cells compared to their respective CD73 KO variants. Left, quantification of geometric mean fluorescence intensity (Geo. MFI). Right, representative histograms showing B2M surface expression. (A-C) Data represent mean ± SD from 3 technical replicates from one individual experiment.

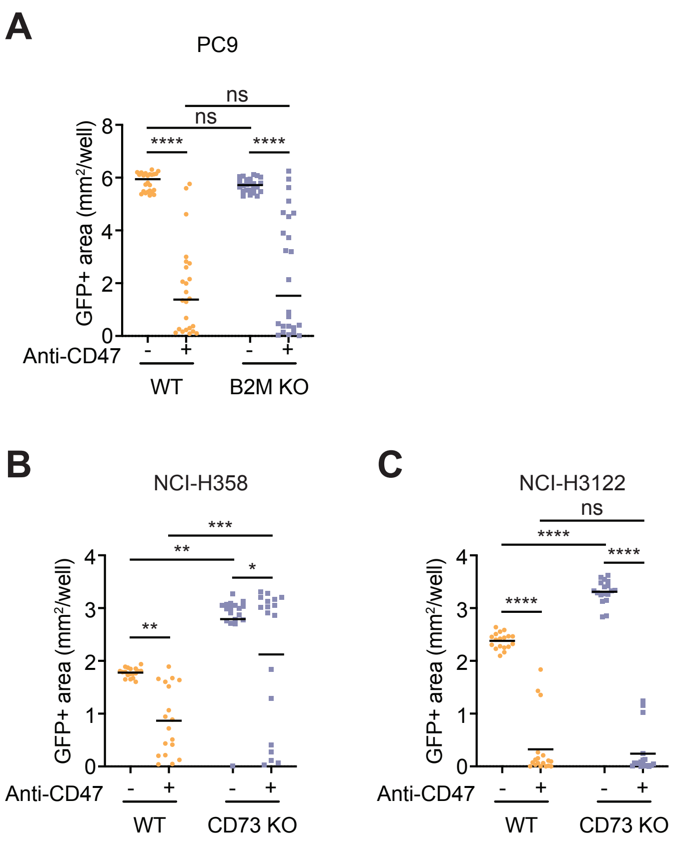

Supplemental Figure 20

Supplemental Figure 20: Genetic deletion of B2M or CD73 does not make some NSCLC cell lines more vulnerable to macrophage attack. (**A**) Evaluation of wild-type versus B2M KO PC9 cells in long-term co-culture assays with human macrophages. Cells were treated with vehicle control or an anti-CD47 antibody. Data are combined from two independent experiments performed with a total of n = 8 independent macrophage donors with 3 technical replicates per donor. (**B-C**) Evaluation of wild-type versus CD73 KO NCI-H358 (B) or NCI-H3122 cells (C) in long-term co-culture assays with human macrophages. Cells were treated with vehicle control or an anti-CD47 antibody. Data represent mean ± SD from n = 6 independent macrophage donors with 3 co-cultures per donor from one experiment. (A-C) ns, not significant, *p<0.05, **p<0.01, ***p<0.001, ****p<0.0001 by two-way ANOVA with Holm-Sidak multiple comparisons test.

Supplemental Table 1: Parameters for fitting plate based titration data for anti-CD47 antibodies with several tyrosine kinase inhibitors to the MuSyC (35) model for multi-drug synergy, 6.5 days after treatment. The MuSyC model parametrically captures synergistic (or antagonistic) effects on efficacy (β), potency (α_{12}, α_{21}) and cooperativity (γ_{12}, γ_{21}). Parameters are only reported if they indicate synergy or antagonism (in parentheses) within the 95% CI. Experiments performed with n = 3-4 independent donors with 3 technical replicates for each of 64 different drug concentration combinations. Donors analyzed separately and combined as indicated.

Treatment	β	α_{12}	α_{21}	γ_{12}	γ_{21}
Gefitinib (combined donors[1])	0.41 (syn.)		5.82 (syn.)		
Gefitinib (donor 239)	0.57 (syn.)	1.70 (syn.)	2.07 (syn.)		
Gefitinib (donor 241)	0.22 (syn.)		15.76 (syn.)		
Gefitinib (donor 242)	0.35 (syn.)				
Osimertinib (combined donors[2])		3.12 (syn.)	8.62 (syn.)		
Osimertinib (donor 246)					4.52 (syn.)
Osimertinib (donor 247)		13.63 (syn.)	18.25 (syn.)		
Osimertinib (donor 248)		6.54 (syn.)		18.04 (syn.)	0.01 (syn.)
Osimertinib (donor 249)			29.15 (syn.)		
Lorlatinib (combined donors[3])	0.28 (syn.)		2.13 (syn.)	3.68 (syn.)	
Lorlatinib (donor 236)			2.07 (syn.)		35.72 (syn.)
Lorlatinib (donor 237)					
Lorlatinib (donor 239)	0.29 (syn.)				
Lorlatinib (donor 240)	0.09 (syn.)		3.52 (syn.)		16.30 (syn.)
Sotorasib (combinded donors[4])	0.84 (syn.)		315.25 (syn.)	0.01 (ant.)	343.55 (syn.)
Sotorasib (donor 199)				3075.50 (syn.)	1105.77 (syn.)
Sotorasib (donor 200)	0.12				
Sotorasib (donor 248)				2.20×10^6 (syn.)	1.20×10^7 (syn.)

[1] Macrophage donors 241 and 242.

[2] All macrophage donors were combined.

[3] All macrophage donors were combined.

[4] Macrophage donors 199 and 200.

Supplemental Table 2: Parameters for fitting plate based titration data for osimertinib with several antibodies to the MuSyC (35) model for multi-drug synergy, 6.5 days after treatment. The MuSyC model parametrically captures synergistic (or antagnonistic) effects on efficacy (β), potency (α_{12}, α_{21}) and cooperativity (γ_{12}, γ_{21}). Parameters are only reported if they indicate synergy or antagonism (in parentheses) within the 95% confidence interval. Replicate 1 was performed using macrophages combined from multiple donors that exhibited a poor response to macrophage-directed therapies. Replicate 2 was performed using a second pool of macrophages that exhibited a standard response to macrophage-directed therapies.

Treatment	β	α_{12}	α_{21}	γ_{12}	γ_{21}
CD47 (mixed donors, replicate 1)					
CD40 (mixed donors, replicate 1)					
PD-L1 (mixed donors, replicate 1)			2.77 (syn.)		
PD-L1 silent (mixed donors, replicate 1)					
CD47 (mixed donors, replicate 2)	0.19 (syn.)	1006.11 (syn.)	7.63 (syn.)		
CD40 (mixed donors, replicate 2)			3.66 (syn.)	39.67 (syn.)	
PD-L1 (mixed donors, replicate 2)	0.32 (syn.)	129.93 (syn.)	20.97 (syn.)		0.57 (syn.)
PD-L1 silent (mixed donors, replicate 2)					

Supplemental Movie 1: Long-term co-culture assays using GFP+ PC9 cells and human macrophages treated with vehicle control. Whole-well imaging was performed of GFP+ channel showing growth of *EGFR* mutant cancer cells in the presence of primary human macrophages (unlabeled). GFP+ cancer cells growth without any substantial inhibition by macrophages. Video depicts 8 days of elapsed time. Images acquired every 4 hours. Scale bar = 1.0 mm.

Supplemental Movie 2: Long-term co-culture assays using GFP+ PC9 cells and human macrophages treated with gefitinib as a single agent. Whole-well imaging was performed of GFP+ channel showing growth of *EGFR* mutant cancer cells in the presence of primary human macrophages (unlabeled). GFP+ cancer cells are restricted in their growth, but persister cells always form and remain in the culture over time. Video depicts 8 days of elapsed time. Images acquired every 4 hours. Scale bar = 1.0 mm.

Supplemental Movie 3: Long-term co-culture assays using GFP+ PC9 cells and human macrophages treated with anti-CD47 as a single agent. Whole-well imaging was performed of GFP+ channel showing growth of *EGFR* mutant cancer cells in the presence of primary human macrophages (unlabeled). GFP+ cancer cells are attacked by macrophages, but foci of cancer cells remain in the culture over time. Video depicts 8 days of elapsed time. Images acquired every 4 hours. Scale bar = 1.0 mm.

Supplemental Movie 4: Long-term co-culture assays using GFP+ PC9 cells and human macrophages treated with the combination of gefitinib and anti-CD47. Whole-well imaging was performed of GFP+ channel showing growth of *EGFR* mutant cancer cells in the presence of primary human macrophages (unlabeled). GFP+ cancer cells are attacked and fully

eliminated by macrophages such that persister cells do not form. Video depicts 8 days of elapsed time. Images acquired every 4 hours. Scale bar = 1.0 mm.
